# Catecholamine precursor modulation of human exploration: Evidence from a large gender-balanced sample

**DOI:** 10.1371/journal.pcbi.1014356

**Published:** 2026-06-11

**Authors:** Angela Mariele Brands, Kilian Knauth, David Mathar, Tim Roedder, Kerstin Lisner, Jan Peters

**Affiliations:** 1 Biological Psychology, Department of Psychology, University of Cologne, Cologne, Germany; 2 Department of Psychiatry and Psychotherapy, University of Bonn, Bonn, Germany; Pantheon-Sorbonne University: Universite Paris 1 Pantheon-Sorbonne, FRANCE

## Abstract

The catecholamine precursor Tyrosine has been linked to improved cognitive performance, but investigations into decision-making and reinforcement learning processes known to be under catecholamine control are sparse. We examined the impact of a single dose of Tyrosine (2g) on reinforcement learning and exploration in a large (n = 63) gender-balanced sample in a within-subjects preregistered study. Reinforcement learning performance was significantly improved under Tyrosine. Based on previous work, we preregistered the hypotheses that Tyrosine would reduce directed exploration, response times, and physiological arousal. However, neither response times nor physiological arousal revealed the predicted reductions. Computational modelling using an established pre-registered reinforcement learning model revealed that the performance improvement under Tyrosine was due to an increase value-driven exploitation, without affecting directed exploration. Non-preregistered modelling analyses then revealed that accounting for higher-order perseveration substantially improved model fit, and substantiated the observation of increased value-driven exploitation under Tyrosine. Furthermore, it revealed reliable reductions in directed exploration and value-independent perseveration under Tyrosine. Tyrosine thus improved reinforcement learning performance by stabilizing choice patterns in the service of optimizing reward accumulation, modulating several computational mechanisms thought to be under catecholamine control.

## Author summary

Catecholamines, such as dopamine and noradrenaline, play an important role in learning from rewards and making decisions. Tyrosine is a building block for these chemicals and sometimes taken as a dietary supplement to improve cognitive functioning. To which extent and how tyrosine may cause these effects is however, largely unclear.

This study investigated the effect of a single dose (2g) of Tyrosine vs. Placebo on a reinforcement learning task in 63 participants (32 self-identified female). In order to tease apart different sub-components of learning and decision-making, we employed a variety of different analysis techniques, including computational modelling.

Contrary to some initial hypotheses, Tyrosine improved performance by increasing value-driven exploitation and decreasing choice behaviour aimed at information gain, while measures of physiological arousal remained unaffected. There were no meaningful differences between male and female participants. These findings indicate that Tyrosine stabilised choice patterns by modulating different computational mechanisms, ultimately leading to optimised reward accumulation.

## Introduction

Freely available supplements that promise enhancing effects on cognition are on the rise. Yet, if and how dietary supplements may affect cognitive function and performance is still poorly understood. Tyrosine (TYR) is the precursor of catecholamine neurotransmitters and is thought to facilitate the synthesis of dopamine, norepinephrine, and epinephrine [1]. Cognitively demanding activities and stress increase catecholamine demand, which in turn leads to a decrease in TYR plasma availability [2,3,4]. Thus, additional intake of TYR is assumed to provide the necessary resources to reach and maintain optimal performance under demanding conditions [2,5].

Such claims seem to be supported by findings on enhancing and protective effects of TYR on working memory (WM) and executive control (especially under conditions of heightened demand; [6,7,2]). Nonetheless, despite the central role of the catecholamine neurotransmitter dopamine (DA) in reinforcement learning (RL) and decision-making [8,9,10, Westbrook et al., 2025,^11^], comparatively little is known about potential TYR-related changes in these functions. An earlier report suggested that TYR may improve some aspects of decision-making [12], but this study was restricted to male participants, thus limiting generalizability. TYR effects on RL and decision-making are of considerable relevance, however, as both aberrant DA-functioning and RL deficits have been linked to several psychiatric disorders and maladaptive behaviours on the subclinical spectrum [13,14,15,16,17,18,19,20]. This resonates with the approach taken in the field of computational psychiatry, which investigates neurocomputational components underlying mental disorders [21,22,17,23,24]. Especially the balance between more elaborate and cognitively costly *goal-directed* choice mechanisms on the one hand and simpler, less flexible *habitual* choice mechanisms, on the other, have emerged as highly relevant. Imbalances between these two broad systems are present across a range of disorders [16,25,26,22, Huys et al. 2021; 17,21], and initial evidence points towards an impact of TYR [12].

A central problem in RL is the tradeoff between exploration (sampling novel options for information gain) and exploitation (selecting known options for reward maximization) [13,27,18,28]. Dynamically changing environments require a balance of these strategies [29,26,28], since both overreliance on exploitation and excessive exploration can lead to performance decrements [30,31,32]. Exploration can be further subdivided into (at least) two component processes: random vs. directed exploration [Gershman, 2018; 2019; 28,33]. While random exploration is characterized by choice randomization, directed exploration is linked to a strategic exploration of uncertain options for information gain [34,31,26,33]. This can be formalized by including an uncertainty-based “exploration bonus” in formal RL models [19,32,20,14,33,35,36,37].

Several mental disorders and subclinical variations thereof have been reliably associated with aberrant explore-exploit behaviour (e.g., [13,38,30, Gershman 2018,^16^,^20^]. Likewise, manipulations of the DA system have been linked to changes in exploration [14,15,39,30,40] and conceptually closely-related model-based control [14,12]. Another factor potentially contributing to dysregulated choice behaviour is perseveration [41,42,43]. Low or high levels of perseveration may be associated with exploitation and exploration, but choice repetition may also occur independent of value and information gain [44]. Such *value-free* perseveration is more closely related to habitual responding, which is linked to various mental disorders [45, Waller et al, 2012,^21^]. Many computational models account for perseveration by including a term reflecting repetition of the previous choice (trial t-1). More recent approaches have extended this to also account for value-free perseveration beyond trial t-1 (i.e., accounting for the impact of longer choice histories) [46,47, Gershman, 2020,^44^].

However, the specific role of TYR in regulating exploration is currently unclear.

To address this issue, we preregistered a counterbalanced, placebo-controlled double-blind within-subject study to assess the impact of a single 2g dose of tyrosine (vs. placebo) in a gender-balanced sample. We opted for a comparatively large sample size (n = 63 in total), considering mostly small samples sizes prevalent in the supplementation field. The preregistration, model files, and data sets can be found at https://osf.io/4z39r/files/osfstorage.

Based on previous research by Mathar et al [12], we formulated the following hypotheses. The first hypothesis (H1) states that we expect a reduction in physiological arousal, namely heart rate (HR) and variance of pupil dilation (PDV) following TYR supplementation. H2 regards the results of another task not presented here. The third hypothesis (H3) includes a) an expected reduction in directed exploration and b) a reduction in RTs under TYR, as well as c) a reduction of DDM parameter α (threshold) under TYR.

## Results

### Model-free results

Descriptive statistics of model-agnostic performance indices separated by factors gender (female vs. male) and condition (PLC vs. TYR) are shown in Fig 1 (see S1 Table). Generally, participants performed above chance, choosing the option associated with the highest reward in 66.57% (SD = 6.39; Fig 1A) of the trials, which earned them an average payout of 19,000 points per session (SD = 1033; i.e., around 63.36 points per trial). Choice data also contained evidence for exploration behaviour, as participants switched to a different option in around 30% of trials (Fig 1B). Median RTs were around.3 seconds (Fig 1C).

**Fig 1:**
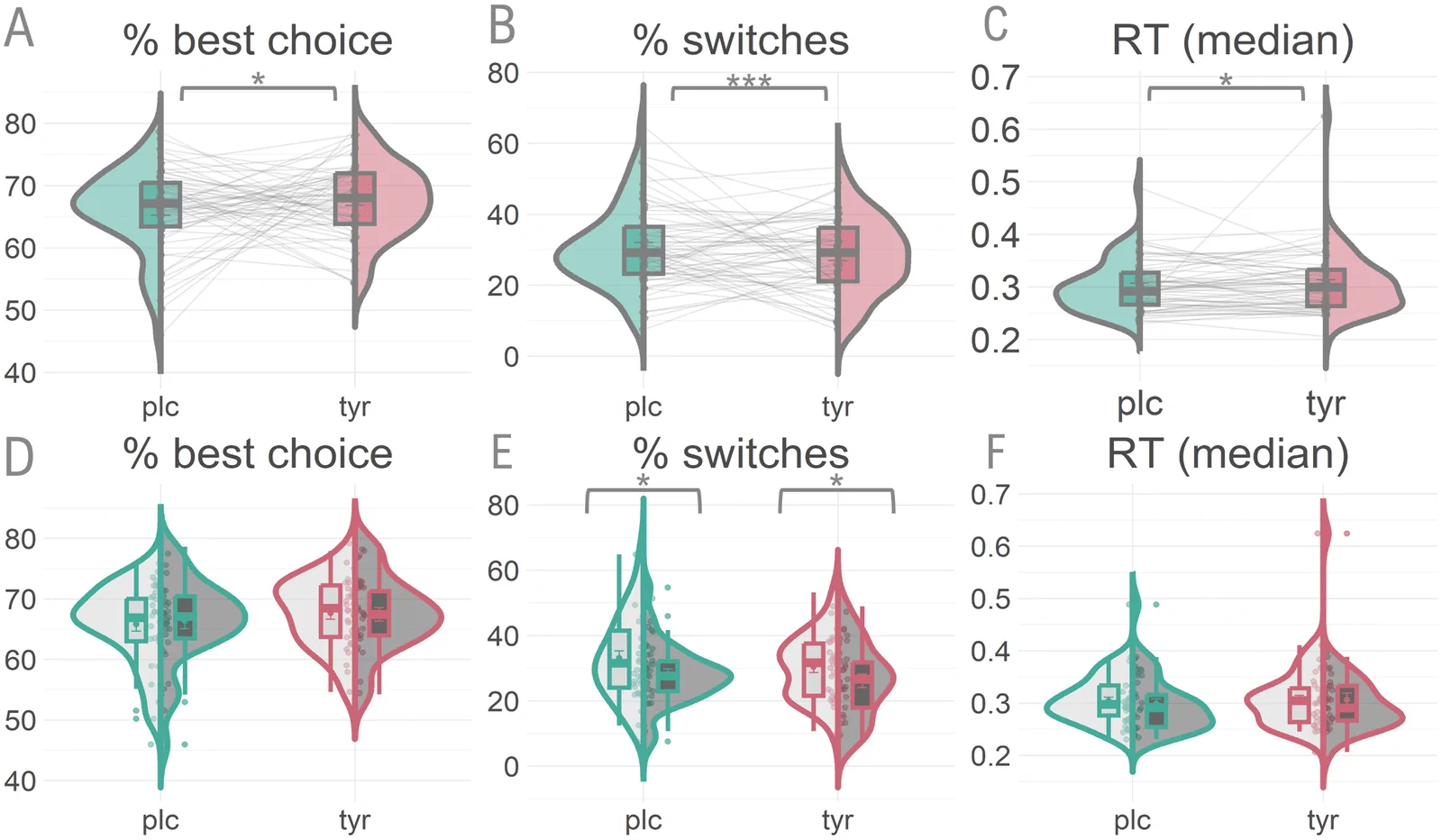
Model-Agnostic Performance Measures Separated by Condition and Gender Group. Supplementation conditions PLC vs TYR are shown in turquoise and magenta, respectively. Dots depict individual subjects. Shading in the lower panel plots mark gender groups (light = female; dark = male). A: percent of choices for the highest-value option. B: proportion of choice alterations (vs. repetitions). C: median reaction times in sec. * p < 0.05 ** p < 0.01 *** p < 0.001.

Trial-wise optimal choices (i.e., choice of the highest rewarded bandit) were increased under TYR vs. PLC) (Table 1; Fig 1A, main effect of condition p = .019), whereas the condition x gender interaction was not significant (Table 1, p > .5).

**Table 1:** Results from Regression Analyses of Model-Free Performance Measures.

	*%best choice*			*RT*			*%switches*		
Predictors	Estimate	CI	p	Estimate	CI	p	Estimate	CI	p
**(Intercept)**	6.60 x10^-1***^	0.65 – 0.67	**<0.001**	3.40 x10^-1***^	0.32 – 0.36	**<0.001**	3.29 x10^-1***^	0.30 – 0.36	**<0.001**
**cond [tyr]**	1.64 x10^-2*^	0.00 – 0.03	**0.019**	4.30 x10^-3^	0.00 – 0.01	**0.045**	-2.39 x10^-2***^	-0.04 – -0.01	**<0.001**
**gend [mal]**	2.50 x10^-3^	-0.02 – 0.02	0.814	-2.73 x10^-3^	-0.03 – 0.02	0.838	-4.65 x10^-2*^	-0.09 – -0.00	**0.032**
cond [tyr] * gend [mal]	-4.69 x10^-3^	-0.02 – 0.01	0.641	2.34 x10^-3^	-0.00 – 0.01	0.449	4.72 x10^-3^	-0.01 – 0.02	0.623

* p < 0.05 ** p < 0.01 *** p < 0.001; cond = condition (plc vs. tyr); gend = self-identified gender (female vs. male).

Switching was significantly reduced under TYR (Table 1, Fig 1B, main effect of condition p < .001), and lower in men vs. women (Table 1, main effect of gender p = .029). The regression on trial-wise RTs also revealed a small increase in RTs under TYR (Table 1) that was in the opposite direction compared to previous RT findings in two other tasks (see [12]).

To gain a basic insight into the influence of past choices on current ones (i.e., perseveration behaviour), we set up a simple regression model, testing the extent to which present choices can be predicted based on choices on the previous four trials [S1 Fig, c.f. Lau & Glimcher, 2005; 44]. All lags indeed showed significant effects on current choices, illustrating a model-agnostic counterpart to the above introduced model-based HOP (i.e., value-free perseveration). Previous choices further remained significant predictors for current choices when additionally including previous rewards in this model (S2 Fig).

### Physiological data

As mentioned previously, we also collected physiological data during both sessions, once at baseline (t0) and a second time prior to task execution (t1). Overall, baseline measures showed high test-retest reliability across both sessions (S3 Fig), with ICC values ranging from 0.76 to 0.96 (S2 Table). Thus, physiological indices exhibited high consistency at baseline. Next, we investigated whether changes in pre- to post-supplementation measurements differed between conditions (i.e., PLC vs. TYR). Pairwise t-tests showed that the percentage of change in HR, HRV, PD, PDV, and SEBR did not significantly vary between supplementation conditions (S4 Fig and S3 Table, all p > .41).

### Model-based analyses

Results from the Bayesian hierarchical modelling approach are structured according to the preregistered general modelling procedure.

### Step 1: Model comparison & selection

Following our preregistration, model comparison was performed separately for each supplementation condition. This was done to clarify whether the manipulation has an influence on model ranking, which was not the case (Table 2). The BL+Bandit model provided the best fit to the data across both conditions (Table 2) and was therefore used for the in-depth analysis of model performance and condition effects on parameters.

**Table 2:** Model Comparison Results Using Leave-One-Out Cross-Validation. BL = Bayesian Learner; QL = Q-Learner; turquoise = PLC; magenta = TYR; elpddiff = difference in estimated log pointwise predictive density; sediff = standard error of the difference.

	PLACEBO			TYROSINE		
model	eldp_diff_	se_diff_	rank	eldp_diff_	se_diff_	rank
BL + Bandit	0.0	0.0	1	0.0	0.0	1
BL + Sigma	-168.6	53.7	2	-258.5	65.5	3
BL + Trial	-237.1	63.2	3	-224.4	56.1	2
QL + Trial	-320.6	68.0	4	-340.0	58.9	4
BL	-572.4	55.1	5	-519.4	48.8	5
QL	-689.1	61.5	6	-674.8	50.6	6
QL + Bandit	-5043.0	282.4	7	-4542.6	237.5	7

The posterior distributions of group-level parameters derived from fitting data from the PLC and TYR condition and corresponding summary statistics of the difference distributions (TYR-PLC) are provided in the supplement (S5 Fig, S4 Table).

To further validate the winning model, we performed a parameter recovery. All parameters showed high correlations between the true and fitted estimates (subject-level parameters: S6 Fig, S5 Table; group-level parameters:S7 Fig).

### Step 2: Modelling of supplementation effects

We next examined a combined model that directly included condition-dependent changes in each parameter due to the supplementation. Here, for each parameter, the PLC condition was modelled as the baseline, and changes in each parameter from PLC to TYR were modelled via additional parameters modelling condition effects (“shift” parameters). Thus, resulting posteriors reflect the *shift* in each parameter from PLC to TYR and directly reflect the effect size in units of that parameter.

Parameters reflecting directed exploration (Fig 2B) and perseveration (Fig 2C) exhibited an estimated change largely centered around 0, reflecting the fact that these choice mechanisms were largely unaffected by TYR supplementation. In contrast, the beta parameter reflecting choice stochasticity (i.e., exploitation) was markedly increased under TYR (Fig 2A; 95%HDI ≥ 0, see Table 3). This dovetails with the model-agnostic results presented earlier: The increase in choices for the highest value bandit and the decrease in switching behaviour can both be attributed to an increase in choice consistency (i.e., an increase in exploitation) under TYR. In contrast, there was no credible evidence for a TYR modulation of directed exploration or perseveration.

**Table 3:** Summary of TYR-Effects on Model Parameters.

Parameter	Median	95% HDI
β_shift_	0.01	[0.00, 0.02]
Φ_shift_	-0.09	[-0.45, 0.34]
ρ_shift_	-0.02	[-1.32, 1.23]

**Fig 2:**
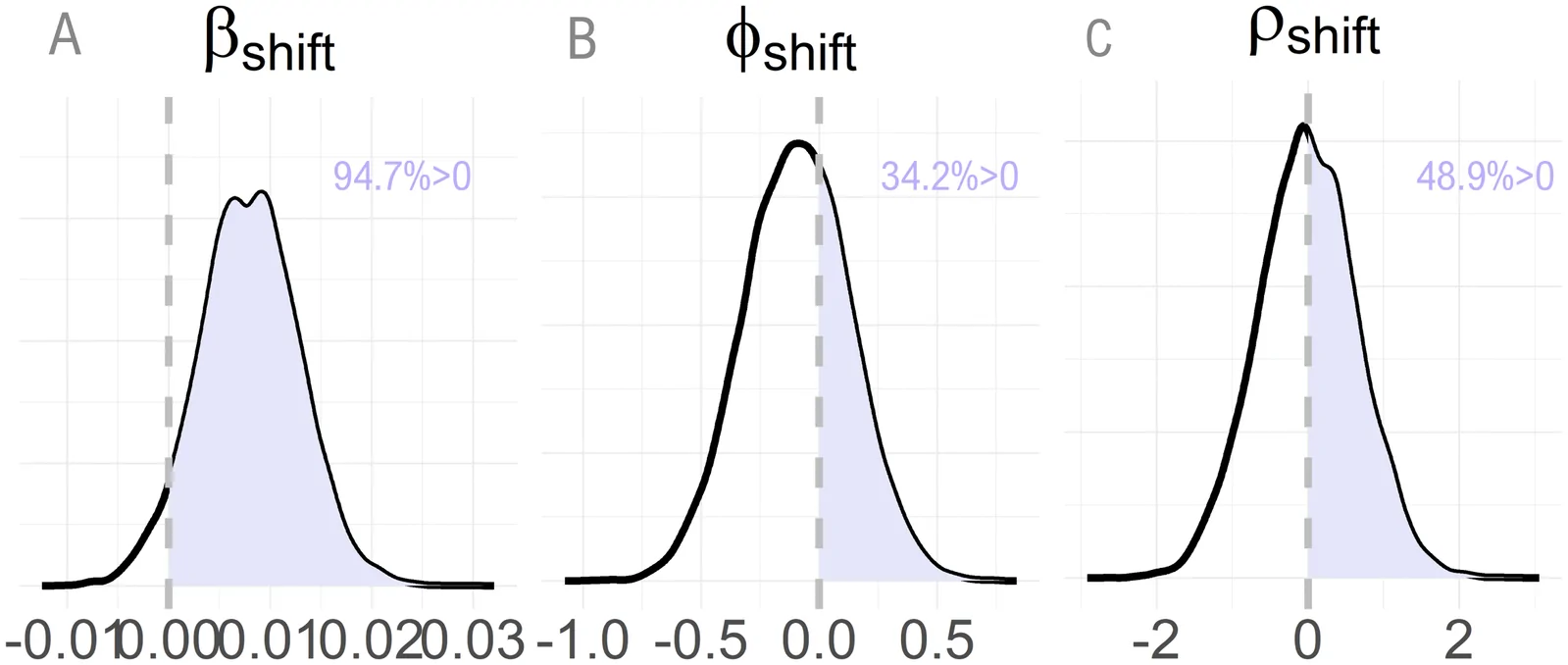
Posterior Distributions of Shift-Parameters Depicting Supplementation Effects. ß: shift (i.e., change) parameter of the SoftMax parameter for value-based choices (i.e., exploitation). Φ: shift of the parameter depicting directed exploration; ρ: shift of the parameter depicting perseveration (choice repetition from t-1). Dashed lines mark a value of 0; percentages show the proportion of the posterior estimates above 0.

To complete the current modelling step, we examined the association of TYR-related changes reported in the section on model-agnostic results with the “exploitation” parameter ß. Correlation analyses (S8 Fig) confirmed that the TYR-induced increase in exploitation (ß) showed the predicted associations with model-agnostic behavioural measures. Higher exploitation was associated with a lower switch rate, and a greater proportion of optimal choices, in both genders.

### Step 3: Investigation of gender differences

We next leveraged our comparatively large and gender-balanced sample to examine potential gender-differences in TYR effects. Due to the sparse research on gender-specific behavioural and neurocomputational changes under TYR, we did not formulate specific *a priori* assumptions on these effects. Here, we applied a *shift* version of the best-fitting model (c.f. Step 2) separately to data from self-identified male and female participants. To examine overall gender effects, we first examined the baseline (PLC condition) parameters per gender (Fig 3). There was no credible evidence for gender effects on any parameter (Fig 3A, 3B & 3C, Table 4). This was further confirmed by a paralleled lack of gender differences in TYR-effects on these parameters (Fig 3D, 3E & 3F).

**Table 4:** Summary of The Posterior Difference-Distribution Between Gender Groups. Parameters denote the group-level means’ difference distribution (fem-mal) of corresponding SoftMax parameters.

Parameter	Median	95% HDI
β_diff_	-0.01	[-0.03; 0.01]
Φ_diff_	0.20	[-0.54; 0.96]
ρ_diff_	-0.89	[-3.10; 1.29]
β_diff-shift_	0.00	[-0.02; 0.02]
Φ_diff-shift_	-0.34	[-1.22; 0.57]
ρ_diff-shift_	-0.33	[-2.91; 2.27]

**Fig 3:**
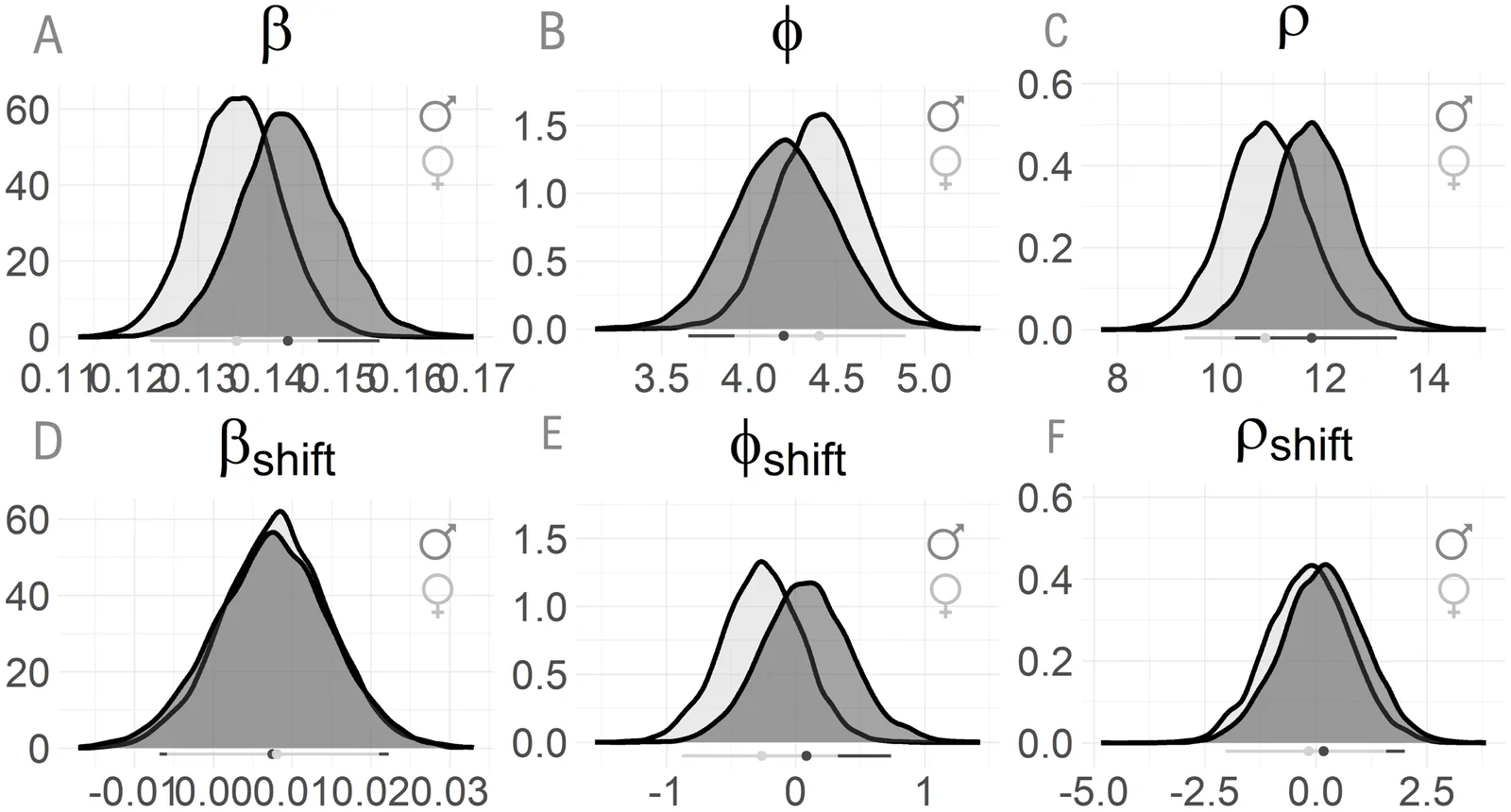
Posterior Distributions for the PLC Condition Separated by Self-Identified Gender Group. A-C: ß: parameter for value-based choices (i.e., exploitation). Φ: directed exploration; ρ: (first-order) perseveration; D-E: TYR-effects on respective model parameters. light and dark gray:results from female and male subsample respectively; bars below the distributions depict the 95% HDI for each group. X-Axis: value estimate of parameter; y-axis: density of the posterior distribution.

Nonetheless, some patterns in the data deserve mention. Numerically, compared to the male sample, female participants showed less exploitation (*stickiness*) and more exploration. These differences may in combination have contributed to the significant gender effect we observed in the model-free analysis of switching behaviour (c.f. section Model-Free Results; Table 1; Fig 1).

### Step 4: Reliability of parameter estimates (Multivariate priors)

Leveraging the comparatively large sample size, we next re-formalized the best-fitting model to obtain a Bayesian estimate of test-retest reliabilities on all model parameters. The hierarchical model was adjusted such that parameters were drawn from bivariate Gaussian distributions across the two supplementation conditions, allowing us to obtain Bayesian estimates of parameter covariance matrices to compute test-retest reliabilities of model parameters. Fig 4 shows the posterior estimates of test-retest correlations for exploitation (β), directed exploration (Φ), and perseveration (ρ). Group-level means for Φ and ρ exhibited high consistency across conditions (r(Φ)=0.59, r(ρ)=0.65), in line with the finding that these parameters were largely unaffected by supplementation. For exploitation (β), on the other hand, the test-retest reliability posterior distribution was symmetrically centred around 0 (r(β)=-0.02), consistent with a considerable change due to the TYR supplementation. More generally, these findings are in line with the idea that exploitation (β) predominantly reflects state-dependent effects, whereas directed exploration (Φ) and perseveration (ρ) also show considerable within-subject stability. These reliability analyses suggest some degree of consistency of parameter estimates across the two supplementation conditions, at least for rho and phi. However, in the present supplementation design, reliability estimates are jointly affected by psychometric properties, and potential condition effects (state-dependency). These estimates should therefore not be taken as “pure” estimates of test-retest reliability.

**Fig 4:**
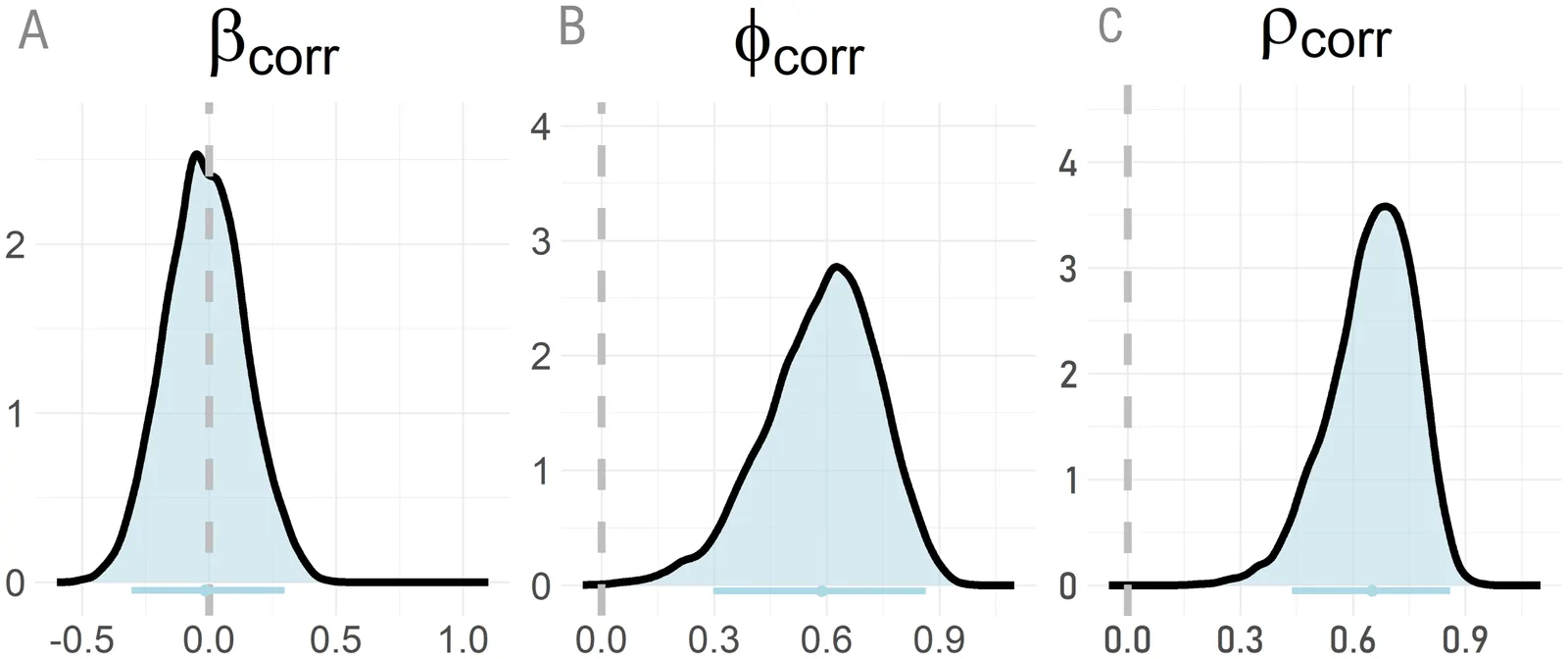
Posterior Distributions of Bayesian Test-Retest Reliabilities. Group-level posterior of the correlation matrices of multivariate model variant (Step 4), showing the test-retest reliabilities (PLC vs. TYR) A: β_corr_, value-based choices (i.e., exploitation); B: Φ_corr_, directed exploration; C: ρ_corr_, perseveration; dashed lines mark x = 0; bars below the distributions depict the 95% HDI; x-axis: value estimate of parameter; y-axis: density of the posterior distribution.

### Step 5: Posterior predictive checks

In a final step, we examined performance of the best-fitting model using posterior predictive checks by simulating choice data based on draws from each participant’s posterior distribution. We deviated slightly from the preregistered approach (which outlined simulating 500 artificial data sets based on the joint posterior group-level parameters) and examined 8000 simulated data sets per subject using the best-fitting model BL + Bandit.

Each simulated data set was based on a subject-specific parameter combination drawn from the participants’ posterior distributions. Each simulated data set was compared to the respective observed data. Median correct choice predictions separated by supplementation condition are shown in Fig 5A. Prediction accuracy was at a similar level for both conditions and well above chance level (PLC: Mdn = 66.31%; TYR: Mdn = 67.54%). We next evaluated, for each participant, each of the 8000 full simulated data sets using model-agnostic measures (% best choice, %switches) and averaged these predicted scores across simulations. These largely reproduced the observed behavioural data in both conditions and both for % best choices (Fig 5B) and switch rate (Fig 5C), although the latter was slightly overestimated by the model. Taken together, posterior predictive checks confirmed that the best-fitting model reproduced key patterns in the data.

**Fig 5:**
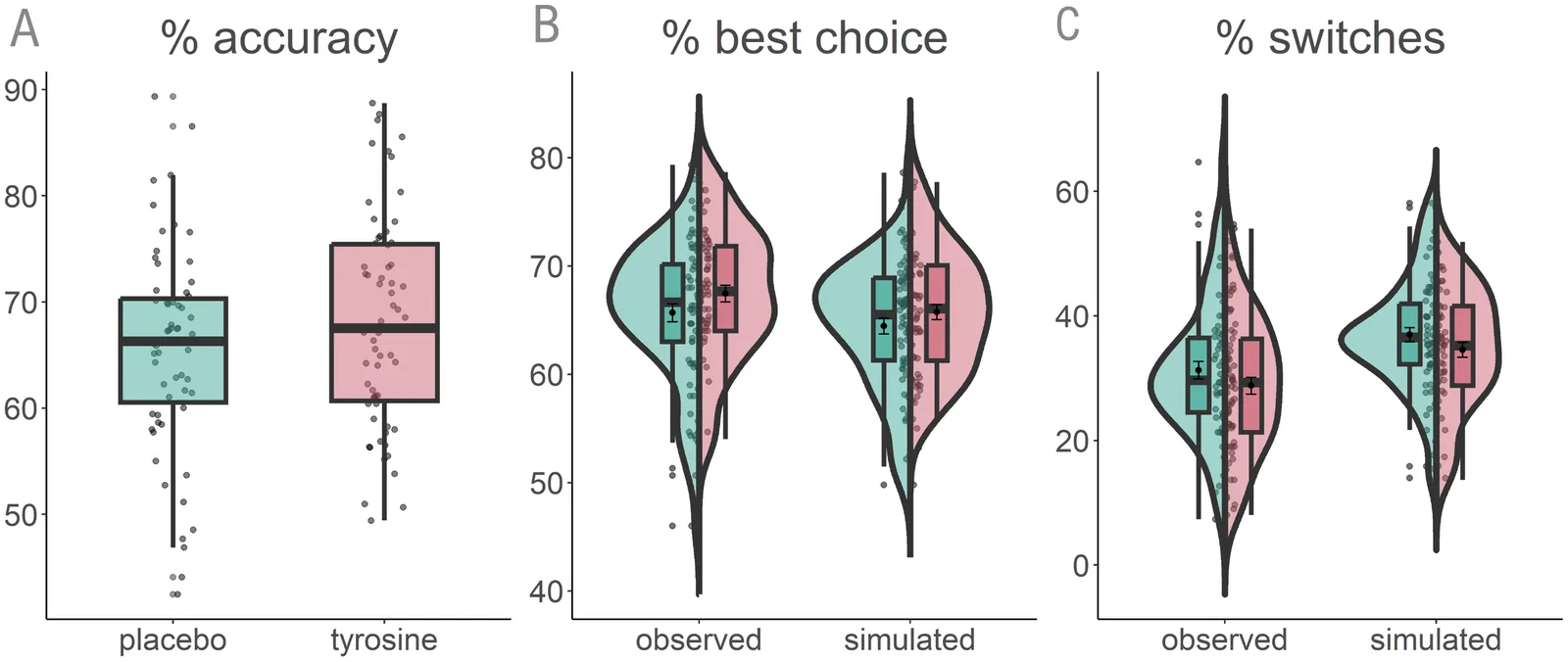
Analysis of Simulated vs. Observed Choice Data. Turquoise = PLC, Magenta = TYR, Points represent participants. A: predictive accuracy calculated as the proportion of correct choice predictions by the winning model compared to observed choices. Panel B & C: Proportion of highest-value choices (%best) and choice alterations (%switches) separated by condition. Observed = empirical data (c.f. model-agnostic results); simulated = predicted choice data based on the winning model.

### Step 6: Exploratory modelling

#### 6.A. Higher order perseveration.

We ran additional, non-preregistered modelling analyses, based on previous results that perseveration behaviour may extend across longer choice sequences [higher-order perseveration; 44,47,37,48]. As mentioned above, we consequently set up another refined version of model BL + Bandit in which the indicator function depicting FOP was replaced by the mechanisms suggested by Miller et al [44], i.e., a *habit vector* ${\text{H}}_{t}$ which tracks the choice history for each bandit (Gershman, 2020; [44]) (see Methods section, S1, S2 & S9 Figs).

This extended model (*Habit;* Table 5) was only applied to the previous BL model variants, which outperformed all QL models; (c.f. Table 1), and we focused on the base model and exploration models BL+Bandit & BL+Sigma. Model comparison favored model variants accounting for HOP, with the extended BL+Bandit model showing the best fit across both conditions.

**Table 5:** Model Comparison Results via Leave-One-Out Cross-Validation. BL = Bayesian Learner; Habit = HOP extension (vs. FOP); turquoise = PLC; magenta = TYR; elpd_diff_ = difference in estimated log pointwise predictive density; se_diff_ = standard error of the difference.

	PLACEBO			TYROSINE		
model	eldp_diff_	se_diff_	rank	eldp_diff_	se_diff_	rank
BL + Bandit + Habit	0.0	0.0	1	0.0	0.0	1
BL + Sigma + Habit	-91.3	61.6	2	-101.1	60.9	2
BL + Bandit	-168.9	36.3	3	-102.2	20.4	4
BL + Sigma	-661.8	95.5	4	-744.1	67.3	5
BL + Habit	-691.0	59.2	5	-613.9	58.1	3
BL	-1193.6	132.7	6	-980.6	77.3	6

As done previously for the FOP version, we also performed a parameter recovery analysis to validate this model version, following the same procedure as before. This yielded satisfactory correlations between true and recovered model parameters (S6 Table, S10 and S11 Figs).

Model recovery showed that for the FOP data sets, both models’ fits were indistinguishable, the HOP model provided a superior fit across all five simulated data sets based on this model (S7 Table).

However, regarding the interpretation of model recovery results, please note that the FOP model is nested in the HOP model (i.e., setting α_HOP_ to 1 yields the FOP formalism). Due to this nested relationship between the models, the HOP model will always be able to fully account for data patterns generated by a FOP mechanism, while the reverse is not the case.

Fig 6 depicts posterior estimates of supplementation effects for the four main parameters of interest (c.f. Results on the BL+Bandit model with the addition of HOP step-size α_HOP_). Modelling results of supplementation effects accounting for HOP reproduced the previously observed increase in exploitation (β) under TYR (Fig 6A and Table 6). However, the extended model also revealed credibly reduced parameters for directed exploration as well as perseveration (Φ and ρ, respectively, 95% HDI < 0; Table 6, Figs 6C & 6D) under TYR. Accounting for HOP therefore led to a more fine-grained decomposition of choice-mechanisms, and now revealed an attenuation in directed exploration and perseveration under TYR as well as substantiating the previously moderate effect on exploitation.

**Table 6:** Posterior Estimates From Step 2 (Shift Model) of the Adapted BL + Bandit + Habit Model; β_shift_ = effect on value-based decision making; Φ_shift_ = effect on directed exploration; ρ_shift_ = effect on perseveration (HOP).

Parameter	Median	95% HDI
β_shift_	0.01	[0.00, 0.02]
Φ_shift_	-0.48	[-1.03, 0.00]
ρ_shift_	-1.85	[-3.64, -0.02]
α_HOP_-_shift_	0.04	[0.00, 0.08]

**Fig 6:**
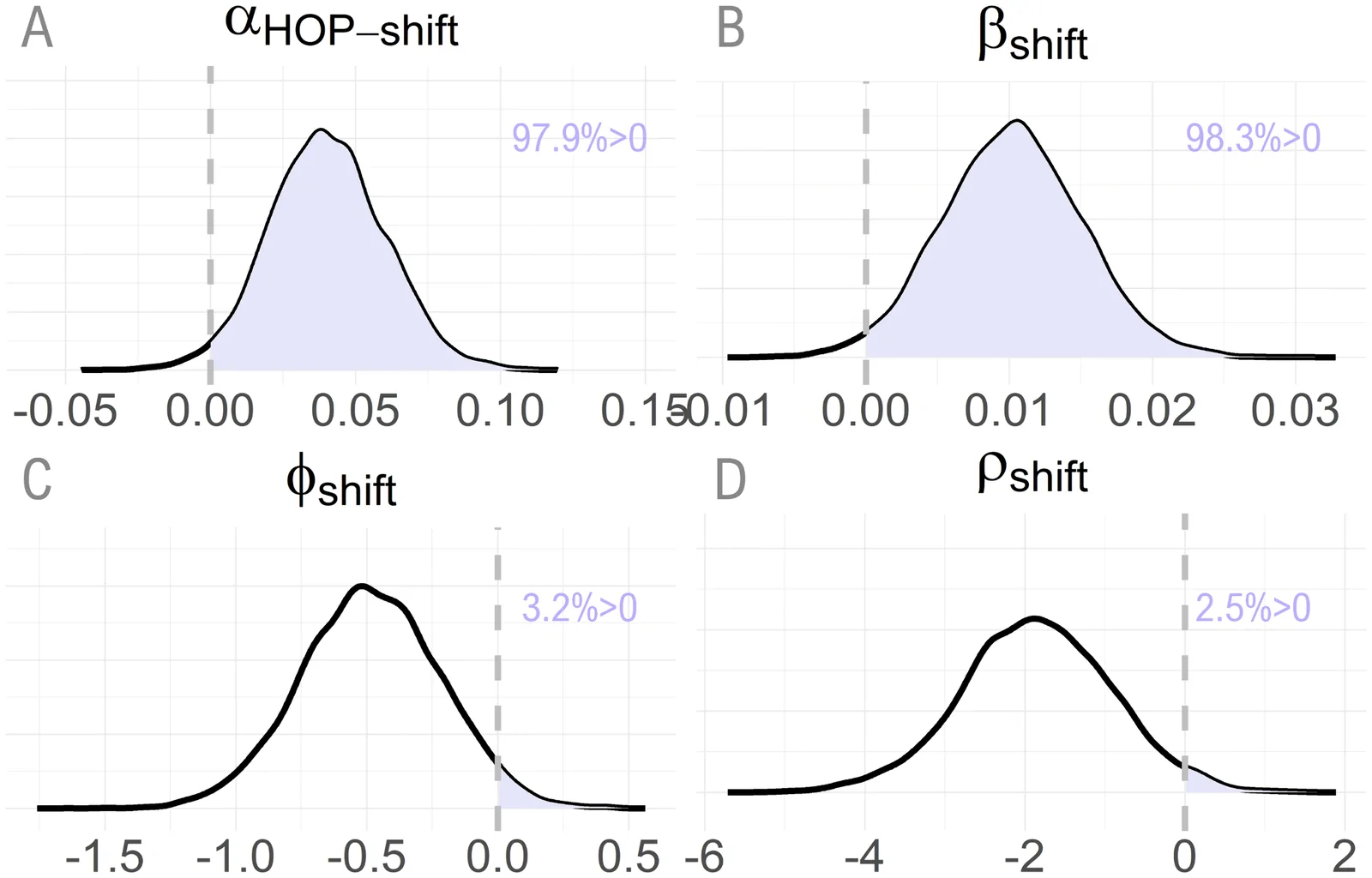
Posterior Distributions of TYR Effects on Parameters from the “Habit” Model-Extension. Depicted are the posterior distributions from Modelling Procedure Step 2 based on the BL+Bandit+Habit Model. “Shift”-Parameters indicate the supplementation effect (PLC vs. TYR) on corresponding parameters: A: higher-order perseveration step-size $\alpha_{HOP-shift}$; B: exploitation $\beta_{shift}$; C: exploration $\Phi_{shift}$; B: perseveration $\rho_{shift}$. Dashed lines mark a value of 0; shaded areas mark values <0.

Results from fitting the extended model to each condition (S8 Table) and gender group separately (S12 Fig, S9 Table) are provided in the supplement.

#### 6.B. DDM: Stay vs. switch choices.

As preregistered we also conducted a model-based analysis of RT-distributions on the bandit task using a Drift Diffusion Model (DDM). In this model each decision is modelled as a noisy evidence accumulation process toward one of two boundaries. These mark the criterion for a decision, so that crossing either *decision boundary* is equivalent to the observable point at which a choice is made [Meyers et al., 2022; 49]. In order to dichotomize the four possible responses on the task, we coded each choice on trial t as either a stay (0) or switch (1) relative to the previous choice on trial t-1. This FOP-categorization yielded the decision boundaries for the DDM-analysis [c.f. 50].

Graphic and tabular depictions of posterior parameter estimates per condition and the resulting difference distributions can be found in the supplement (S13 Fig, S10 Table). Modelling of TYR effects on DDM parameters in a combined model indicates that non-decision time ($\tau_{shift}$) and boundary separation ($\alpha_{shift}$) were largely unaffected. HDIs of the a priori bias ($z_{shift}$; 95% HDI=[-0.01, 0.05]) and the rate of evidence accumulation (δ_shift_; 95% HDI=[-0.45, 0.04]), show more noteworthy deviation from 0 while, however, still not providing credible evidence for an effect (Fig 7, Table 7).

**Table 7:** Posterior Parameter Estimates Derived from a DDM With Stay-Switch Decision-Boundaries. α = boundary separation; z = bias; δ = drift rate; τ = non-decision time.

Parameter	Median	95% HDI
α_shift_	0.01	[-0.02, 0.04]
z_shift_	0.02	[-0.01, 0.05]
τ_shift_	0.002	[-0.01, 0.01]
δ_shift_	-0.20	[-0.45, 0.04]

**Fig 7:**
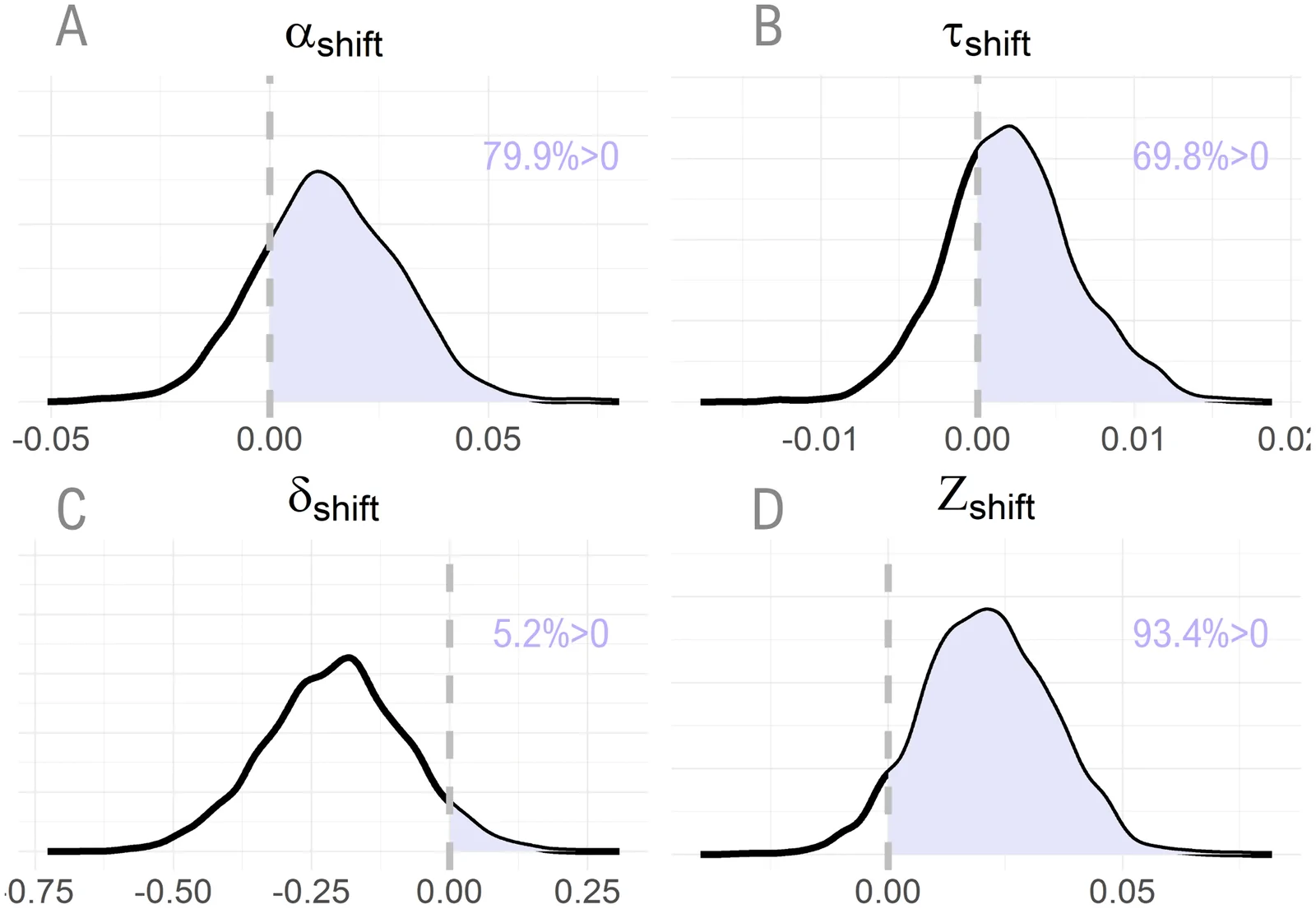
Posterior Distributions of DDM-Parameters Depicting Changes Due To Condition. Group-level shifts in parameter estimates from condition PLC to TYR;A: α = boundary separation; B: τ = non-decision time; C: δ = drift rate; D: z = bias. Decision Boundaries were defined as 0 = stay (i.e., repetition of preceding choice); 1 = switch (choice alteration).

The above reported model-free slowing effect of TYR on median RTs thus, may be driven by contrasting effects: a small shift of the starting point in favor of a choice switch (increased z, Fig 7D) paired with a simultaneous increase in the drift toward a choice repetition (decreased δ, Fig 7C). Recall, however, that this analysis was based on the dichotomization into *stay* and *switch* choices, while the referred to main effect of RT-slowing under TYR did not make this distinction.

Thus, overall examination of the combined model including condition effects on DDM parameters (Fig 7, Table 7) revealed no credible evidence for TYR-related effects.

With the aim to provide further insight into the relation between DDM parameters and model-agnostic RT measures, we conducted correlation analyses. Here, we looked at the relationship between the supplementation change in parameters and difference in mean RTs for stay and switch trials (S14 and S15 Figs). The tyrosine-related shift in the drift parameter (Mdn = -0.20;Table 7) showed significant positive association with the difference (TYR-PLC) in mean RTs for switch trials (r = 0.45, p < .001) as well as the difference in proportion of switch choices (r = 0.95, p < .001). These findings are consistent with the overall model specification, such that lower drift rates indicated a trend toward stay decisions (i.e., lower switch rates). Model-agnostic findings of lower switch rates under tyrosine also fall in line with this logic (i.e., stronger drift toward stay-decision under tyrosine is mirrored in behavioural readout).

## Discussion

Here we show that a single dose of the catecholamine precursor TYR modulates reinforcement learning and decision-making in a commonly applied RL task with a dynamic reward schedule. Most notably, we found an overall increase in choice consistency and optimal choices under TYR, and both effects were largely independent of gender. Contrary to our preregistered hypotheses, we found no changes in physiological arousal (H1) and slightly increased (vs. decreased) RTs (H3b) following TYR supplementation. Comprehensive computational modelling further revealed that TYR supplementation improved performance by increasing value-based exploitation, in line with the observed increase in optimal choices and a reduction in the switch rate. In this preregistered model, the predicted reduction in directed exploration (H2) was not confirmed. Extending this model variant by also accounting for higher-order perseveration (HOP) substantiated the increase in value-based exploitation. In this model, TYR furthermore reduced directed exploration and value-independent perseveration, effects that were potentially masked in the previous, simpler model variant. This was accompanied by a change in HOP step-size, indicating a reduced integration window of prior choices under TYR. The reduction of directed exploration in the extended model is in line with H3a. In an exploratory DDM analysis of stay vs. switch decisions, we found TYR supplementation to influence subjects’ starting points and drift rates, rather than lead to a reduction in thresholds, as stated in hypothesis H3c.

These results provide insights into the neurocomputational mechanisms underlying TYR supplementation effects on RL performance. Model comparison revealed that behaviour was best accounted for by a model that assumes an uncertainty-dependent learning process (i.e., Bayesian Learner via Kalman Filter). The best-fitting model further included a heuristic-based directed exploration mechanism (i.e., “Bandit Heuristic”, where participants track how many other options were sampled since a specific option was last selected), as in related work [47]. These modelling results are in line with previous studies that also favored Kalman Filter over less flexible and uncertainty-naïve Q-learning variants [see, e.g., 14; Daw et al., 2006; 20]. However, in the present study, directed exploration was better accounted for by a heuristic process, as opposed to the uncertainty estimate of the Kalman Filter model [c.f. e.g., 14; Daw et al., 2006, 31].

Analysis of this preliminary best-fitting model revealed that the performance improvement under TYR was due to an increase in value-driven exploitation (beta parameter of the softmax function) [51,52,53]. Parameters reflecting (heuristic-based) directed exploration and perseveration (FOP) did not exhibit meaningful variation across conditions based on this model variant, although numerical trends pointed toward a reduction in these two components under TYR. Extending this model by accounting for HOP in an additional exploratory modelling step, however, revealed a substantial improvement in model fit. It also substantiated the previously observed increase in value-based exploitation under TYR, whereas TYR-related reductions in directed exploration and value-independent perseveration became considerably more substantial. Our pre-registered hypothesis of a reduction in directed exploration under Tyrosine was thus confirmed only in this extended model, which, however, showed a substantial improvement in model fit. It remains an open question if Tyrosine effects on these different computational processes result from a shared underlying mechanism. In the current study however, Tyrosine-related shifts in parameters depicting HOP step-size, exploitation, and directed exploration exhibited no significant correlations between each other (S11 Table), pointing toward separate mechanisms.

Both the preregistered model and the extended model exhibited good parameter recovery [54]. Model recovery showed that data generated using a higher-order perseveration process where substantially better accounted for by the respective model. In contrast, for data generated by a first-order perseveration process, model fit was similar for both models. This is to be expected in the case of nested models, as the HOP model will always be able to account for a FOP process by setting α_HOP_ to 1, resulting in the simpler nested FOP model.

This effect of model refinement echoes previous findings of improved granularity upon defining and formalizing related processes [55,54]. For example, Chakroun et al. [14] observed an increase in directed exploration estimates after accounting for FOP, compared to a model variant without a perseveration term [Daw et al., 2006], which dovetails with the effects of the HOP extension in the current study. Specifying previously unaccounted-for processes can thus aid the identifiability and interpretability of other model components by accounting for regularity in the data that would otherwise reflect unexplained variation. Finally, it should be noted that not all parameter changes under Tyrosine necessarily reflect the performance improvement observed for the model-agnostic analyses. For example, both excessive and reduced directed exploration may impair performance, depending on task demands [36]. The model-agnostic performance improvement therefore reflects the net result of overall Tyrosine effects across model parameters.

The work by Chakroun et al. also relates to the present results with respect to the role of DA, as it involved a more direct manipulation of DA levels via a pharmacological intervention with either Haloperidol or Levodopa (LDOPA; vs. PLC). Based on their computational model, L-DOPA reduced directed exploration compared to both placebo and the D2 antagonist Haloperidol. Although a direct comparison is complicated by the different approaches to catecholamine modulation (pharmacological vs. supplementation-based), these results converge with our observation of reduced directed exploration under TYR in the best-fitting model variant. TYR supplementation presumably elicits a more indirect manipulation of DA-levels compared to pharmacological approaches [56,2,57,58,7]. Nonetheless, the observed reduction in directed exploration observed here dovetails with the results by Chakroun et al. [14], while the robust increases in exploitation under Tyrosine across models appear somewhat distinct from the Chakroun et al. [14] results.

Mathar and colleagues [12] followed the same supplementation regime as done here but examined a different RL-task (two-step task; [59]). Although this complicates a direct comparison of results, it may explain contrasting RT effects of TYR in Mathar et al. and the present study. Mathar et al. observed RT reductions under TYR for the two-step task, whereas we observed RT increases for the bandit task, and this might be related to the specific decision problem. The two-step task is more complex due to its sequential, probabilistic architecture, and involves binary decisions on all stages, whereas participants decide between four options on the bandit task. Demands therefore clearly differ between tasks. Across both studies and tasks, however, participants exhibited a higher tendency to repeat vs. alternate preceding actions following the ingestion of TYR, consistent with the idea of choice stabilization. Generally, TYR effects may manifest differently depending on the task demands and performance-related processes.

In contrast to our earlier study [12], we did not observe effects of tyrosine supplementation on any of the physiological measures recorded. Note that this was also the case when analyzing male participants only (S12 Table), which rules out that differences are due to the different gender distributions across studies [12, tested only males]. At the same time, we replicated the findings of Mathar et al. [12] with respect to test-retest reliability of physiological measures (assessed at t0, prior to ingestion of tyrosine or placebo). Reliability was > .70 for all measures, showing that the failure to replicate the physiological effects is unlikely to be due to poor data quality or poor reliability. Since the overall experimental procedures were highly consistent across the two studies, the failure to replicate the previous physiological effects might be due to a false positive effect and/or sample specific effects in the earlier smaller study.

In order to provide more complete perspective on the observed data, we also modelled RT-distributions for choice switches vs. repetitions using a variant of the drift diffusion model (DDM).This analysis, independent of further assumptions regarding learning and valuation processes, revealed a supplementation effect on the drift rate parameter, reflecting a tendency for an increased rate of evidence accumulation (in favour of choice-repetitions under TYR. However, an opposing effect was found for the bias (starting point), which was shifted towards the “switch”- boundary. These contrasting effects may partially explain the small yet significant increase in median RTs under TYR (vs. PLC) observed in our model-agnostic analyses.

The present study focused on effects of a single TYR dose, and did not investigate long-term effects of supplementation. While there are first reports indicating positive long-term effects of TYR on domains such as working memory, and fluid intelligence [60], more exhaustive investigations are clearly required. Such future work would preferably also include more diverse samples. Another aspect that would be of interest in the context of longitudinal studies in light of our results (exploratory modelling using HOP) concerns perseveration and habitual responding. Because a key differentiating factor between perseveration and habits concerns their temporal extension, long-term investigations might shed light on TYR effects on perseveration and/or habitual responding over time. While perseveration can to some extent be measured in a cross-sectional laboratory-based studies, habits are much harder to capture as they have developed over and may persist across more extended time periods [46; see also 61,62].

There are several potential modes of action of a low dosage supplementation regime as implemented in the present study [see, e.g., 2,Jongkees et al., 2020; 58,7]. A common interpretation of TYR effects is based on the assumption that increasing precursor availability (e.g., via supplementation) can increase neurotransmitter synthesis under demanding conditions [Attiepoe et al., 2015;^56^,^2^,^3^], However, individual supplementation effects may depend on individual differences in the DA system [63], which may contribute to mixed effects in the literature. Furthermore, the degree to which catecholamine neurotransmitters beyond DA may be affected by TYR remains unclear. Even when focusing on the role of DA in RL, as probed by, e.g., the restless bandit task applied here, supplementation may affect phasic and/or tonic DA levels [64,65,63] and effects may additionally depend on individual factors such as baseline availability and synthesis capacity [see, e.g., 56,7].

Overall, current findings are in line with recent theories on the role of DA in reinforcement learning and decision-making (Niv et al., 2007; Friston et al., 2012; Mikahel et al., 2021). The theory of *Rational Inattention* for example, posits that precision in learning and reliance of actions on learned representations (e.g., reward magnitudes) heavily rely on individuals’ dopaminergic functioning. Mikahel and colleagues (2021) state that high (vs. low) tonic DA levels foster learning from rewards (vs. punishment or low reward) and influence action control in favour of exploitation (vs. exploration). Constituent mechanisms here may include a more pronounced contrast in subjective values (due to more precise estimates) and a resulting preference for higher value options (i.e., exploitation over exploration) [see also 66]. Thus, under the assumption that TYR supplementation in the present study modulated DA-tone, our results largely align with these ideas.

## Limitations & future directions

Despite the relatively large and gender-balanced sample and comprehensive modelling procedures, several limitations of our approach need to be addressed. Conclusions are limited to western, educated, white and cis-gendered, mostly young and able-bodied volunteers, limiting generalizability. We also did not examine potential dosage effects, but used a widely-used fixed dosage of 2g [see, e.g., 2,67,68,69,12]. Recommendations and applications with regard to TYR-doses vary across previous investigations (e.g., Deijen 2005: 14mg/kg), which precludes direct comparison of findings and may contribute to mixed effects. However, control analyses of model-agnostic effects that included weight as an additional random effect confirmed our results (S13 Table). Nonetheless, even a more direct and rigorous tracking of catecholamine levels would not eliminate variability of TYR ingestion via other exogenous sources, such as dietary choices. While for the current study, we did ask participants to abstain from large, protein-rich meals in advance of testing sessions, adherence to this request could not be directly controlled. It should be noted though, that despite their potential influence on present findings, TYR-related effects found were stable across gender groups as well as age and weight ranges (as narrow as these may have been).

Due to the experimental design and nature of the supplementation procedure, potential dopaminergic and noradrenergic effects cannot be clearly disentangled. However, based on previous research, DA appears to be critically involved in directed exploration. Research using the D2/D3 receptor antagonist amisulpride shows that DA modulates sensitivity to choice-relevant features such as reward values, depletion rates, and opportunity costs [15]. Further studies revealed that DA mediates exploration by changing the precision of value-based choice selection, such that increased DA activity is associated with decreased exploration and vice versa [70]. In line with these findings, Chakroun et al [14] found DA to attenuate directed exploration and modulate neural representations of uncertainty in the insula and dorsal ACC. Noradrenaline, while also regulating exploratory behaviour, seems to be more closely associated with random exploration [71]. When participants receive ß-adrenergic antagonist propranolol, they showed reduced tendency to use value information and switched more while rewards were still high [15]. Additional research confirms that random exploration is attenuated under propranolol but not under DA blockade, demonstrating that this form of exploration is more closely linked to noradrenergic influences [30]. NA activity seems to influence exploration by changing outcome sensitivity, and this effect seems to be sex-dependent [70]. The lack of differences in physiological arousal between conditions PLC and TYR further points to a dopaminergic over noradrenergic effect. While the cardiovascular system may also be influenced by DA, it is primarily under autonomic control and thus, more closely associated with NA effects [72]. Pupil dilation is even more closely related to LC-NA functioning and sympathetic activity, and not primarily DA [73, Joshi et al., 2015; 74]. While the SEBR is commonly used as a proxy for DA function [Jongkees & Colzato, 2016], previous findings are mixed and include reports of missing associations [75,76]. Together, these results may be more compatible with a dopaminergic as opposed to a noradrenergic effect.

Although our results do provide new insights into latent effects of TYR on cognitive subprocesses, our study design only allowed for a cross-sectional snapshot of such effects. Investigations into long-term effects of TYR (self-) administration on (among others) learning and decision-making are called for (see above). This seems especially important as the practical application of such supplements is not limited to a one-time low-dose ingestion. Indeed, between 40–60% of customers in India (and around 45% in the United States) reported daily use of dietary supplements in a recent survey [Rakuthen 77,78].

As outlined previously, the exact neurochemical effects of TYR are still only partially understood, but are thought to be modulated by baseline DA levels (see, e.g., prominent related work by [2] on a potential inverted-U shaped association of DA and executive functions). Consequently, effects of additional exogenous manipulation of catecholamine synthesis (e.g., via supplementation) may elicit different effects depending on such endogenous factors.

Another limitation may lie in the analysis approach applied. Despite their clear advantages and benefits compared to model-free compound measures, cognitive computational models have their own inherent limitations. Model variants considered in this project cover a considerable range of established accounts, but all conclusions remain restricted to the examined model space. For example, even the best-fitting model only accounted for around 67% of choices, on average, such that there is clearly room for further improvements. We wish to address and tackle this issue head-on by making our analysis procedure, including computational details along with explicit model codes available. Other interested researchers are invited to test and tweak modelling accounts presented here, and report on their results obtained in this endeavor.

## Conclusion

We investigated the effect of a single dose of the catecholamine precursor tyrosine (2g) on RL and decision-making in a young and healthy gender-balanced sample. Tyrosine improved performance in terms of the percentage of optimal choices, and extensive computational analyses linked this effect to an increase in value-based exploitation. Further model extensions that accounted for higher-order choice perseveration effects [44,47,37,48] substantiated this effect, and further clarified underlying mechanisms by revealing a reduction in directed exploration (resonating with previous pharmacological studies, [14]) and value-independent perseveration under TYR. In a comparatively large and gender-balanced sample, our results reveal robust effects of TYR on neurocomputational processes thought to be under dopaminergic control.

## Methods

### Ethics statement

The study procedure was approved by the ethics committee of the Faculty for Human Sciences of the University of Cologne (approval number: JPHF0149).

### Sample

We pre-registered a sample size of 68 (34 subjects per group). Overall, 69 subjects completed participation, four of which were excluded due to high BDI-scores and the fifth participant was excluded due to their acute self-reported health status (i.e., no sleep, high caffeine intake; see exclusion criteria as preregistered). We further excluded one more participant who showed nonsensical response patterns in one of the sessions. Thus, a total of N = 63 (32 self-identified female) comprised the final sample. Participants had a mean age of M = 23.38 (SD = 5.30; range = 18–52 years). The vast majority (62 out of the final 63) had a degree in higher education and an average Body-Mass-Index (BMI; M = 22.93, SD = 3.60; range = 17.53 to 34.72). A descriptive summary of the self-reports is provided in the supplement (S14 Table).

### Procedure

Participants participated in testing at the University of Cologne Biopsychology lab on two separate days. Sessions were spaced 3–5 days apart and took place in the same time slot each for every participant. Prior to participation all subjects gave their written, informed consent. On each testing day we first obtained baseline measures of physiological measures such as skin conductance response, heart rate and pupillometry. Individuals then consumed 200ml of orange juice containing either 2g of dissolved TYR (TheHut.com Ltd.) or PLC (microcrystalline cellulose), followed by a 60-minute waiting period in a separate room (with minimal external stimulating or distracting features; i.e., empty office room). Participants were instructed to abstain from drinking alcohol or consuming protein-rich food from the evening prior and up to each testing day in order to minimize external influences on TYR levels [Deijen, 2005].

Following the 60min waiting period, physiological measures were obtained again, and participants performed a short temporal discounting task (10–15min). Temporal discounting and physiological data were obtained following the procedures of Mathar et al. [12] and were included as a direct replication, which will be reported elsewhere. This was followed by 300 trials of a four-armed restless bandit task [14,20, Daw et al., 2006] with changing option reward values over time (Fig 8). Values changed independently following Gaussian random walks. Participants could gain an additional payout of up to 4€ depending on task performance. Task and supplementation order were randomized and counterbalanced within each gender group and thus, also across the entire sample. At the end of the second testing day participants also completed a number of self-reports and sociodemographic questionnaires not relevant for the present report (see preregistration, S14 Table).

**Fig 8:**
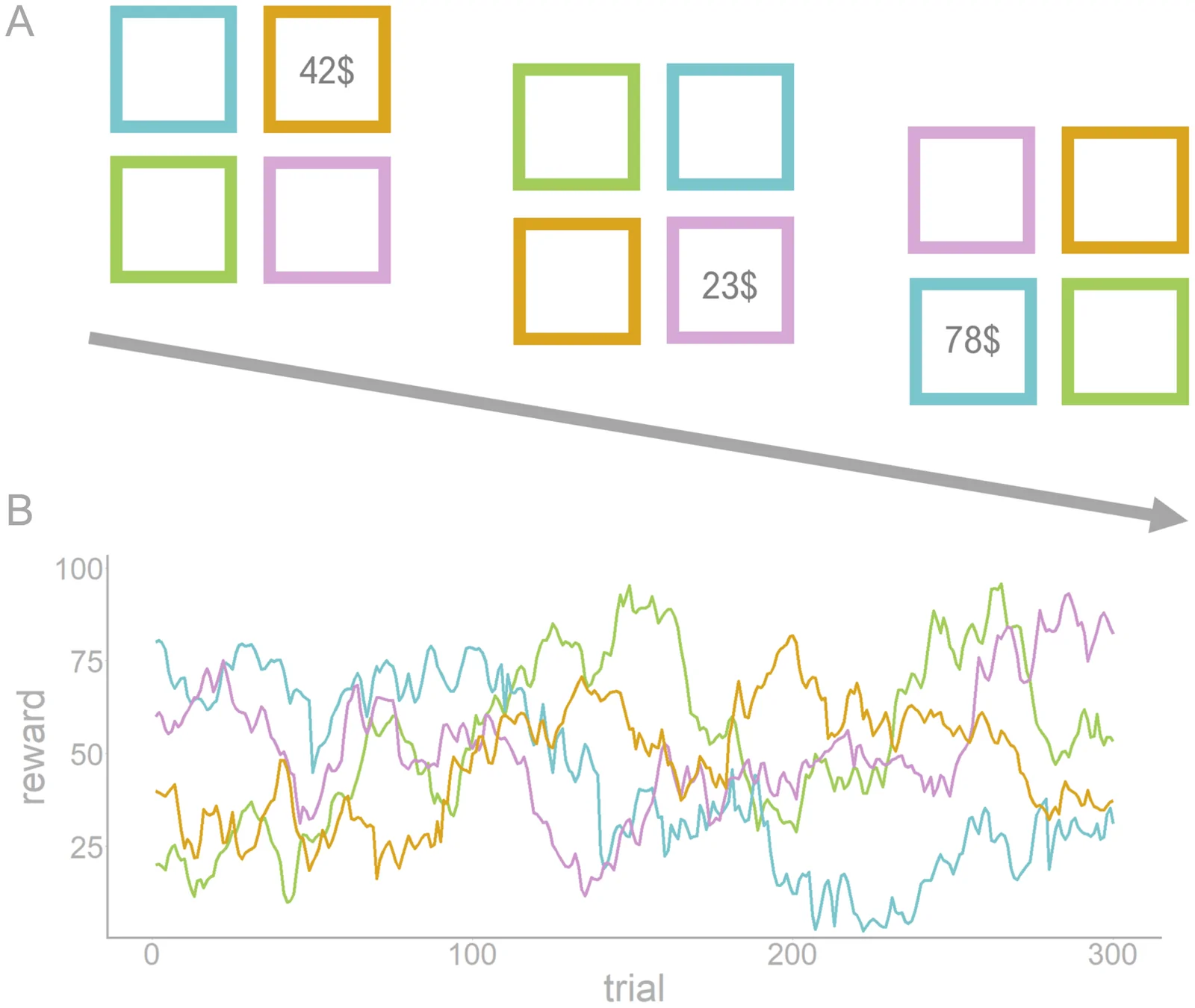
Schematic Depiction of the 4-Armed Restless Bandit Task. On each trial, participants selected between four choice options marked by different colors. Following each choice, the reward associated with that option was displayed. Rewards varied independently according to Gaussian random walks [20; Daw et al., 2006; 14] and were displayed in the form of hypothetical monetary amounts between 0 and 100€.

### Model-agnostic behavioural data analysis

As model-agnostic variables of interest, we examined median reaction times (RTs), the proportion of highest-value choices (%optimal), and participants’ tendency to switch between choice options (%switches). To this end, we set up linear mixed models with factors gender (male vs. female), condition (tyrosine vs. placebo) and their interaction as fixed effects. Subjects were included as random effects. This yielded three separate regression models for RT, %optimal, and %switches. Regression analyses using linear mixed models included gender and supplementation condition, along their interaction as fixed and subjects as random effects using the *lmerTest* package [79] in the statistical program R [80].

### Physiological data

As mentioned previously, we also collected physiological data during both sessions, once at baseline and a second time prior to task execution (i.e., after supplementation and waiting period). While we did not originally plan to include these measures in our current report, we followed reviewers’ suggestions and did perform some basic analyses. This was done primarily in order to further illuminate on dopaminergic and/or potential noradrenergic effects. Physiological measures encompassed heart rate (HR in bpm), HR variability (HRV) indexed by the SD of normal-to-normal intervals (SDNN in ms), pupil dilation (PD) and it’s variability (PDV), as well as the spontaneous eye blink rate (SEBR in blinks per minute). A description of the apparatuses and exact procedure can be found in the preregistration.

ECG data were preprocessed using NeuroKit2 package [81] in Spyder (version 6.1; [82]), yielding mean HR (in bpm) and SDNN (in ms) per subject, session, and timepoint. Pupil measures (PD & PDV) were processed in R [80] using packages *zoo* [83] *dplyr* [84], and *signal* [85]. This again, resulted in mean estimates for PD and PDV per subject, session, and timepoint. SEBR was assessed by averaging counts of eye blinks over a five minute period, manually assessed by two independent raters. In parallel fashion to Mathar and colleagues [12], we evaluated two main variables: 1) test-retest reliability of baseline measurements and 2) percentage of change in physiological measures from pre- to post-supplementation timepoints per condition. These two variables thus, provide indication for potential changes in sympathetic functioning (and/or their stability/robustness) in light of supplementation.

### Hierarchical bayesian modelling

In addition to the analysis of model-agnostic performance indices (see above) our main focus was on computational modelling using a hierarchical Bayesian approach (c.f. preregistered general procedure). In a first step, we compared a range of competing computational accounts of behaviour, based on commonly-applied RL model variants [see, e.g., 14; Daw et al., 2006; 54,47]).

### Computational models

Computational model variants differed with regard to their implementation of value updating (i.e., learning) and directed exploration effects.

We compared models based on a classic TD learning algorithm SARSA [QL; 86], such that Q-values of each option i on trial t are updated according to:

(1) $Q_{i,t+\text{1}}=\;Q_{i,t}+\;\alpha \delta_{t}$

with the reward prediction error (RPE):

(2) $\delta_{t}={\;r}_{i,t}\;-\;Q_{i,t}$

and a constant learning rate α (ranging from 0 to 1).

The alternative learning component included uncertainty-dependent updates, which change on a trial-wise basis using a Kalman Filter [Kalman, 1960]:

(3) ${\hat{\mu }}_{\text{i},\text{t}}^{\text{post}}={\hat{\mu }}_{\text{i},\text{t}}^{\text{pre}}+\;{\text{k}}_{\text{t}}\delta_{\text{t}}$

Here ${\hat{\mu }}_{i,t}^{post}$ denotes the posterior (i.e., updated) belief about the reward of option i, which is updated using the prior belief ${\hat{\mu }}_{i,t}^{pre}$ and RPE $\delta_{t}$, scaled by the Kalman gain $k_{t}$, with:

(4) $\delta_{\text{t}}=\;{\text{r}}_{\text{t}}-{\hat{\mu }}_{\text{i},\text{t}}^{\text{pre}}$ and
(5) ${\text{k}}_{\text{t}}=\;{\hat{\sigma }}_{\text{i},\text{t}}^{\text{2pre}}/\;({\hat{\sigma }}_{\text{i},\text{t}}^{\text{2pre}}\;+{\hat{\sigma }}_{\text{o}}).$

As can be seen in Eq. 5, the Kalman gain depends on two different sources of variance, ${\hat{\sigma }}_{i,t}^{\text{2}pre}$ depicts the variance associated with the reward value of option i, while ${\hat{\sigma }}_{o}$ is the observation variance. This parameter indicates the noisiness of observations (vs. the variation in the true underlying reward distribution). For a more exhaustive account, we refer the reader to the preregistration and previous work [see, e.g., 14; Daw et al., 2006; 87].

These two learning mechanisms (QL & BL) were combined with different variants of a basic SoftMax function to yield action probabilities. In the two base models (simply denoted QL & BL) choice probabilities were modelled according to:

(6) $P(a_{i,t}=a)=\;\frac{exp\left[\beta (Q_{i,t}+{pb}_{i,t})\right]}{\sum_{j}exp\left[\beta (Q_{j,t}+{pb}_{j,t})\right]}.$

Note that for readability Q(a) is used for both learning mechanisms, with $Q(a)={\hat{\mu }}_{i,t}^{post}$ for the BL model variants.

In order to investigate and compare different exploration mechanisms, we tested three variants of an uncertainty bonus. The *classic* implementation assumes that participants update an internal belief model which mirrors the underlying Gaussian random walks (for more details see preregistration; see also, e.g., Daw et al., 2006; [14]). This version was only combined with the BL base model, as the QL model does not assume participants to represent and update a full environment model (as is the case in Bayesian accounts). This yielded model *BL + Sigma* (c.f. Table 1), where the *exploration bonus* (${\text{eb}}_{i,t}$) simply consists of subjects’ (model-based) variance estimates (i.e., beliefs) ${\hat{\sigma }}_{i,t}^{\text{2}pre}$ scaled by a free directed exploration parameter ϕ, such that:

(7) ${\text{eb}}_{i,t}=\;\phi *\;{\hat{\sigma }}_{i,t}^{\text{2}pre}$

In addition, we also considered two different counter-based uncertainty proxies [see 47] which are computationally less costly and may therefore be more parsimonious. In one variant (“trial heuristic”) we assume participants to simply track, for each bandit, the number of trials since these have last been sampled, yielding uncertainty proxy $U_{T_{i}}$, implemented in models QL + Trial and BL + Trial (c.f. Table 1). In another variant (“bandit heuristic”), we assumed participants to track the number of alternative options that have been sampled since an option was last selected (i.e., ranging from 0 to 3), yielding uncertainty proxy $U_{B_{i}}$, implemented in models QL + Bandit and BL + Bandit (c.f. Table 1). The resulting exploration bonus for either heuristic was defined analogous to the classic version shown in Eq. 7 above:

(8) ${\text{eb}}_{i,t}=\;\phi *\;U_{B_{i,t}}.$

In all models we also accounted for simple perseveration behaviour, defined as repeating the choice from the preceding trial (see also [14]). To this end we defined a *perseveration bonus* as:

(9) ${\text{pb}}_{i,t}=\;\rho *\;{\text{I}}_{\text{i},\text{t}},$ where ${\text{I}}_{\text{i},\text{t}}$ is an indicator function which equals 1 if the previous choice (t-1) is repeated and 0 otherwise.

For all models apart from the base versions (c.f. Eq. 6 above) choice probabilities were modelled according to:

(10) $P\left(a_{i,t}=a\right)=\frac{exp\left[\beta (Q_{i,t}+{eb}_{i,t}+{pb}_{i,t})\right]}{\sum_{j}exp\left[\beta (Q_{j,t}+{eb}_{j,t}+{pb}_{j,t})\right]}.$

### Exploratory modelling

In addition to the preregistered model variants, we considered one further adaptation of the previously described SoftMax model, as well as a Drift Diffusion Model (DDM).

#### Higher order perseveration.

For the SoftMax extension, we incorporated a Higher Order Perseveration (HOP) term, replacing the Fist-Order (FOP) analogue described above. HOP assumes that current choices are not only influenced by the just preceding one (i.e., FOP), but instead are biased by the subject’s choice history reaching further back in time. This process is formalized in the form of a record that stores prior choices independent of any (subjective) reward-values assigned to them [44,48].

This *habit vector* ${\text{H}}_{t}$ is, analogous to subjective reward values of choice options, updated using a decay rate (since we are looking back instead of planning ahead – but can be seen as akin to learning rate parameters). Trial-wise updates of habit strength ${\text{H}}_{i,t}$ for option i on trial t follow:

(11) ${\text{H}}_{i,t}=\;{{\text{H}}_{i,t-\text{1}}+\alpha }_{HOP}*\;({\text{I}}_{\text{i},\text{t}}-H_{i,t-\text{1}}),$

where ${\text{I}}_{\text{i},\text{t}}$ is the same indicator function used in the previous FOP model variants (${\text{I}}_{\text{i},\text{t}}$ equals 1 if a choice is repeated and 0 otherwise; c.f. Eq. 9 above). The decay-rate $\alpha_{HOP}$ updates the habit strength associated with each choice option and determines the extent of the temporal integration window [see also 44]. An exemplary depiction of the influence of varying $\alpha_{HOP}$ values can be found in the supplement (S9 Fig).

Action probabilities follow the same SoftMax as in Equation 10 above, with the only difference that ${pb}_{i,t}$ is defined as:

(12) ${\text{pb}}_{i,t}=\;\rho *\;{\text{H}}_{i,t}.$

#### Drift diffusion model.

Next to the models using the SoftMax function to model action probabilities, here we also implemented a Drift Diffusion Model (DDM). As DDMs depict the decision process as a noisy accumulation of evidence towards one of two decision boundaries, we dichotomized choices from the four-armed bandit task by coding choices as either repetitions (*stay*) or alterations [*switch,* 50].

Due to their wide applicability and adaptability DDMs have been of great utility to several research foci, including the study of disease-relevant cognitive processing. Aberrant evidence accumulation has thus, been proposed as another candidate for a transdiagnostic neurocomputational phenotype and risk factor [Sripada & Weigard, 2021]. Transcending the realm of clinical applications, several research groups have successfully refined, adapted combined this basic DDM architecture with other common computational models, e.g., of intertemporal choice [12,88,66] or as the decision rule in dual-choice RL tasks [e.g., 89,12,90].

For the current study, and as this is an exploratory addition to our more exhaustive/extensive computational modelling approach presented above, we have limited the DDM- analyses of choice and RT data from the 4-armed restless bandit task on the basic version (i.e., without modifications accounting for learning and subjective valuation).

Basic versions of this model commonly encompass the following key components involved in the decision-process prior to border crossing: the *drift rate* (δ), which as the name suggests is a parameter indicating the speed by which evidence is accumulated and thus is negatively associated with the overall RT. The *boundary separation* (α) parameter can be interpreted in light of response caution as it regulates the amount of evidence needed until a choice is made (the decision criterion; so that larger values have a prolonging effect of overall RTs; [49; 91]). The parameter z determines a potential a priori bias. If the two possible decisions (as was done here) are defined as [0 1], z=0.5 would denounce a neutral starting point, while values closer to either boundary denote an according bias (i.e., here values <0.5 would indicate a bias toward choice repetition and >0.5 would indicate one toward switching). Typically, DDMs further account for *non-decision time* with parameter τ, which depicts not further specified (e.g., perceptual and motoric) processes that also contribute to response latencies.

In addition to the preregistered RT-preprocessing (exclusion of upper & lower 1% on the group-level and 2.5% on the subject-level), we further set an absolute lower cutoff of 150ms for DDM fitting.

Using the *RWiener* package [Waberisch & Vandekerckhove, 2014] RTs were modelled according to:

(13) ${RT}_{t}\sim \;wfpt(\alpha ,\;\tau ,z,\delta ).$

Here the *Wiener First Passage Time* (wfpt) describes trial-wise RTs depending on the subject-specific boundary separation α, non-decision time τ, an initial bias z, and the drift rate δ.

### Hierarchical bayesian modelling in STAN

All models were formulated in the STAN modelling language and were fit using the package *RStan* using the Rstudio interface [80,92]. Each model was fit using Markov-Chain-Monte-Carlo (MCMC) via the no-U-turn sampler (NUTS), with four chains running 10000 iterations each, 8000 of which were discarded as warm-up. Ȓ is a measure of convergence across chains, indicating the ratio of between-chain to within-chain variance. Here, values of Ȓ ≤ 1.1 were considered acceptable.

Subject-level parameters were assumed to stem from a shared group-level Gaussian distribution, so that for each model parameter x, the hierarchical models also contained hyperparameters M_x_ and SD_x_ modelling group-level means and standard deviations. For all M_x_ and SD_x_, we set low informative priors assuming them to follow a normal (M = 0, SD = 10) and uniform (Min = 0, Max = 20) distribution, respectively. Subject-level parameters were set to be drawn from this resulting group-level normal distribution (M_x_ and SD_x_). All parameters with constraints [0,1] were estimated in standard normal space, and back-transformed to their original range within the model.

Prior distributions for the DDM were likewise low informative and are provided in the supplement (S15 Table).

#### Model comparison.

For the comparison of candidate models we used the *loo* package [93], which calculates the estimated log pointwise predictive density (elpd), providing an index for the predictive accuracy of a model via leave-one-out cross-validation. Here, the superior model in a given set of candidate models therefore has an elpd difference value of 0, and all competitor models are then ranked according to their distance (in elpd) to the best-fitting model (lower elpd_diff_ values indicate a higher relative ranking).

#### Model validation.

To further validate the winning model, we performed a parameter recovery. To this end, we drew five samples from the posterior distribution of (subject-level) model parameters from the single-condition fit based on the PLC data. These estimates were then used to simulate choices for the full sample (N = 63, 300 trials). Parameter estimates yielded from fitting these simulations are subsequently compared to the true, underlying estimates. Thus, high correlations between true and recovered parameters indicate good recoverability [54,36].

In a similar vein, we performed a model recovery analysis for the winning RL model and it’s respective exploratory extension. In this step, we fitted the simulated data sets from the parameter recovery analyses (i.e., winning model & exploratory model) to both model variants [54,36].

## Supporting information

S1 Fig Influence of Past on Present Choices. (PDF)

S1 Table Summary Statistics of Self-Report Measures and Basic Demographic Information. (PDF)

S2 Fig Influence of Past Choices and Rewards on Current Behaviour. (PDF)

S2 Table Intraclass Correlations of Physiological Baseline Measures. (PDF)

S3 Fig Reliability of Physiological Baseline Measures. (PDF)

S3 Table Difference in Physiological Changes Between Conditions. (PDF)

S4 Fig Changes in Physiological Measures Due to Supplementation. (PDF)

S4 Table Summary of The Posterior Difference-Distribution. (PDF)

S5 Fig Posterior Distributions of Group-Level Mean Parameters Derived From the Winning Model (BL + Bandit). (PDF)

S5 Table Correlation of True vs. Recovered Parameter Estimates from Model BL + Bandit. (PDF)

S6 Fig Parameter Recovery Results for the BL+Bandit Model. (PDF)

S6 Table Correlation of True vs. Recovered Parameter Estimates from Model BL+ Bandit+ Habit. (PDF)

S7 Fig True and Recovered Group-Level Parameter Estimates Based on Model BL+Bandit. (PDF)

S7 Table Model Comparison for Model Recovery. (PDF)

S8 Fig Association of Task Performance Measures with Supplementation-Effects on Value-Based Decision-Making. (PDF)

S8 Table Posterior Summary Statistics of Group-Level Parameters from Model BL + Bandit + Habit. (PDF)

S9 Fig Exemplary Habitual Controller Values. (PDF)

S9 Table Posterior Summary Statistics of TYR-Effect Parameters Based on Model BL + Bandit+ Habit. Separated by Gender Group.
(PDF)

S10 Fig Parameter Recovery Results from Model BL+ Bandit+ Habit. (PDF)

S10 Table Single Condition Posterior Summary Statistics of DDM Fits. (PDF)

S11 Fig True and Recovered Group-Level Parameter Estimates Based on Model BL+ Bandit+ Habit. (PDF)

S11 Table Correlations Between TYR-Effects on BL+ Bandit+Habit Parameters. (PDF)

S12 Fig Posterior Distributions from the Exploratory Model Extension Separated by Gender Group. (PDF)

S12 Table Difference in Physiological Changes Between Conditions for Male Subsample. (PDF)

S13 Fig Posterior Distributions of DDM-Parameters Separated by Condition. (PDF)

S13 Table Control Regression Models Predicting Model-Agnostic Performance Indices While Accounting for Weight and Age. (PDF)

S14 Fig Mean Reaction Times For Stay vs. Switch Decisions. (PDF)

S14 Table Overview of Self-Report Instruments. (PDF)

S15 Fig Correlations Between DDM-Parameters and Model-Agnostic Measures. (PDF)

S15 Table Priors for DDM Hyper-Parameters. (PDF)
