# Supplementary material for: Catecholamine precursor modulation of human exploration: Evidence from a large gender-balanced sample
Source: PLoS Comput Biol. 2026 Jun 11;22(6):e1014356. doi: 10.1371/journal.pcbi.1014356 (PMC13719043; doi:10.1371/journal.pcbi.1014356)
Supplement: Supplementary file 1 [file pcbi.1014356.s001.pdf]

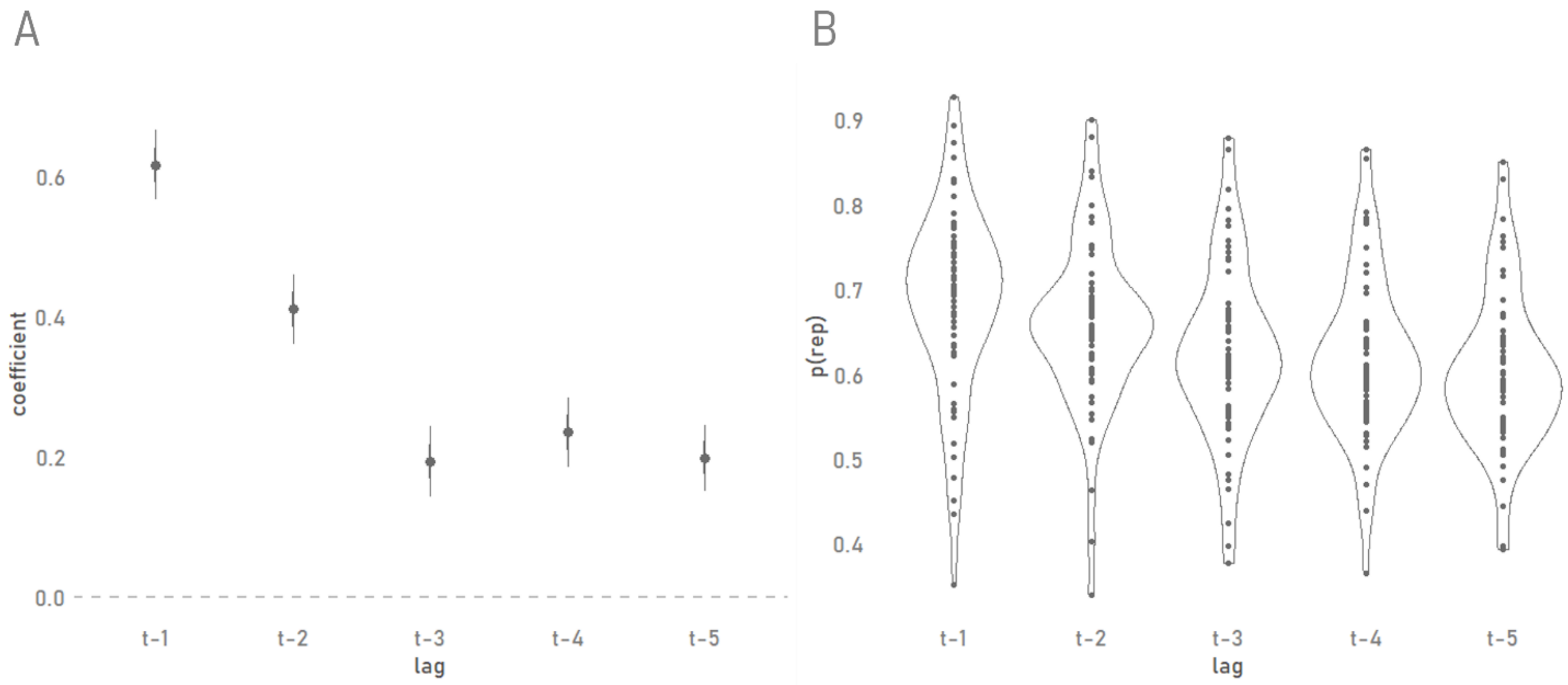

S1 Fig. Influence of Past on Present Choices. A: Regression coefficients (y-axis) of choices one to five trials prior (x-axis) when predicting current choices. Points depict the point estimate of regression coefficients, lines indicate the 95% confidence interval of these estimates. All predictors were highly significant ($p<.001$). B: Probability of repeating choices (y-axis) from one to five trials prior (x-axis). Points depict data from individual subjects.
