# Supplementary material for: Catecholamine precursor modulation of human exploration: Evidence from a large gender-balanced sample
Source: PLoS Comput Biol. 2026 Jun 11;22(6):e1014356. doi: 10.1371/journal.pcbi.1014356 (PMC13719043; doi:10.1371/journal.pcbi.1014356)
Supplement: Supplementary file 2 [file pcbi.1014356.s002.pdf]

S1 Table. Summary Statistics of Self-Report Measures and Basic Demographic Information.

	fem (N=32)	mal (N=31)	Total (N=63)	p value
**age**				0.018
Mean (SD)	21.844 (2.529)	24.968 (6.799)	23.381 (5.296)	
Range	18.000 - 29.000	19.000 - 52.000	18.000 - 52.000	
**AUD**				0.137
Mean (SD)	5.469 (3.398)	6.806 (3.655)	6.127 (3.563)	
Range	0.000 - 14.000	0.000 - 14.000	0.000 - 14.000	
**BIS15**				0.033
Mean (SD)	29.438 (5.847)	32.581 (5.596)	30.984 (5.896)	
Range	20.000 - 42.000	21.000 - 44.000	20.000 - 44.000	
**BDI**				0.712
Mean (SD)	6.875 (5.167)	6.419 (4.552)	6.651 (4.840)	
Range	0.000 - 17.000	0.000 - 16.000	0.000 - 17.000	
**BIS**				0.006
Mean (SD)	19.969 (2.265)	18.452 (1.964)	19.222 (2.239)	
Range	16.000 - 24.000	13.000 - 23.000	13.000 - 24.000	
**BASrew**				0.589
Mean (SD)	16.188 (1.891)	15.935 (1.788)	16.063 (1.831)	
Range	13.000 - 20.000	12.000 - 19.000	12.000 - 20.000	
**BASdrive**				0.785
Mean (SD)	12.281 (1.836)	12.161 (1.635)	12.222 (1.727)	
Range	8.000 - 16.000	8.000 - 15.000	8.000 - 16.000	
**BASfun**				0.791
Mean (SD)	11.938 (1.999)	12.065 (1.788)	12.000 (1.884)	
Range	7.000 - 15.000	7.000 - 14.000	7.000 - 15.000	
**ZTPfut**				0.080
Mean (SD)	3.817 (0.454)	3.628 (0.389)	3.724 (0.430)	
Range	2.923 - 4.615	2.923 - 4.308	2.923 - 4.615	
**ZTPpresF**				0.666
Mean (SD)	3.438 (0.403)	3.394 (0.400)	3.416 (0.399)	
Range	2.200 - 4.200	2.200 - 4.100	2.200 - 4.200	
**ZTPpresH**				0.020
Mean (SD)	3.196 (0.311)	3.410 (0.393)	3.302 (0.367)	
Range	2.571 - 3.929	2.357 - 4.214	2.357 - 4.214	
**ZTPpastP**				0.769
Mean (SD)	2.480 (0.589)	2.524 (0.587)	2.502 (0.583)	
Range	1.500 - 3.625	1.250 - 4.125	1.250 - 4.125	
**ZTPpastN**				0.008
Mean (SD)	2.352 (0.490)	2.724 (0.585)	2.535 (0.566)	
Range	1.545 - 3.545	1.545 - 3.818	1.545 - 3.818	
**weight**				< 0.001
Mean (SD)	64.781 (11.727)	78.065 (11.422)	71.317 (13.293)	
Range	50.000 - 108.000	58.000 - 110.000	50.000 - 110.000	
**income**				0.016
Mean (SD)	616.000 (455.338)	1077.806 (942.233)	843.238 (766.427)	
Range	0.000 - 1900.000	0.000 - 3300.000	0.000 - 3300.000	
