# Supplementary material for: Catecholamine precursor modulation of human exploration: Evidence from a large gender-balanced sample
Source: PLoS Comput Biol. 2026 Jun 11;22(6):e1014356. doi: 10.1371/journal.pcbi.1014356 (PMC13719043; doi:10.1371/journal.pcbi.1014356)
Supplement: Supplementary file 3 [file pcbi.1014356.s003.pdf]

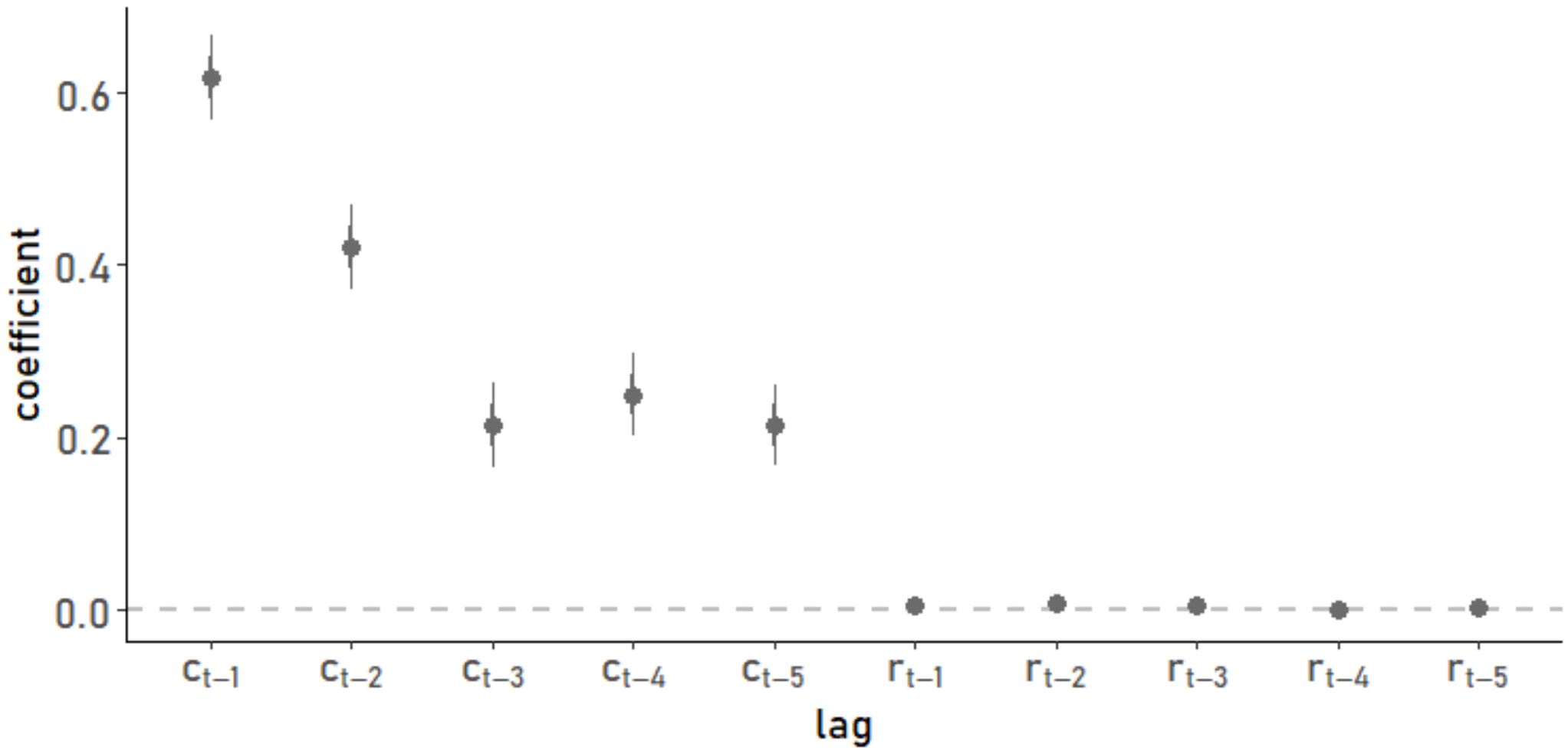

S2 Fig. Influence of Past Choices and Rewards on Current Behaviour.

The plot depicts results from a regression analysis predicting current choice based on choices and rewards of the past 5 trials. The x-axis depicts the different predictors: c_{t-x} indicated the choice made x trials ago and r_{t-x} indicates the reward received x trials ago. The y-axis depicts the estimated value for each predictor.
