# Supplementary material for: Catecholamine precursor modulation of human exploration: Evidence from a large gender-balanced sample
Source: PLoS Comput Biol. 2026 Jun 11;22(6):e1014356. doi: 10.1371/journal.pcbi.1014356 (PMC13719043; doi:10.1371/journal.pcbi.1014356)
Supplement: Supplementary file 4 [file pcbi.1014356.s004.pdf]

S2 Table. Intraclass Correlations of Physiological Baseline Measures.

measure	ICC [95% CI]
heart rate (HR)	.76 [.60 - .85]
heart rate variability (HRV)	.80 [.67 - .88]
pupil dilation (PD)	.96 [.94 - .98]
pupil dilation variability (PDV)	.91 [.85 - .95]
spontaneous eye blink rate (sEBR)	.83 [.72 - .90]

Note. ICC: Intraclass Correlation. 96%CI: 95% confidence interval.
