# Supplementary material for: Catecholamine precursor modulation of human exploration: Evidence from a large gender-balanced sample
Source: PLoS Comput Biol. 2026 Jun 11;22(6):e1014356. doi: 10.1371/journal.pcbi.1014356 (PMC13719043; doi:10.1371/journal.pcbi.1014356)
Supplement: Supplementary file 5 [file pcbi.1014356.s005.pdf]

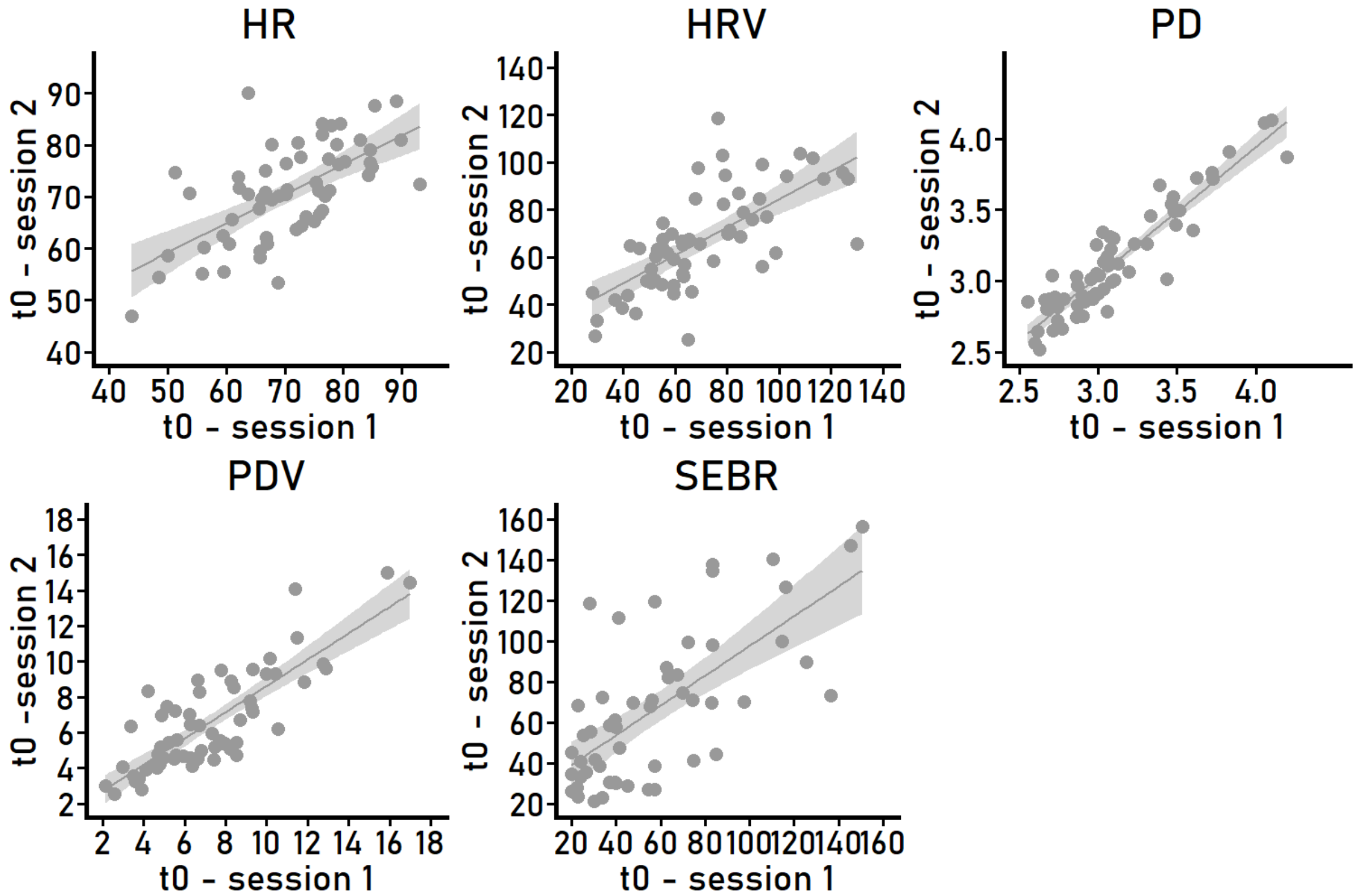

S3 Fig. Reliability of Physiological Baseline Measures. HR: heart rate, HRV: HR variability, PD: pupil dilation, PDV: variance in PD, SEBR: spontaneous eye blink rate. X-axes display baseline (time t0) measurements from the first, and the Y-axes from the second session. Points depict individual subject and grey areas show the standard error.
