# Supplementary material for: Catecholamine precursor modulation of human exploration: Evidence from a large gender-balanced sample
Source: PLoS Comput Biol. 2026 Jun 11;22(6):e1014356. doi: 10.1371/journal.pcbi.1014356 (PMC13719043; doi:10.1371/journal.pcbi.1014356)
Supplement: Supplementary file 6 [file pcbi.1014356.s006.pdf]

S3 Table. Difference in Physiological Changes Between Conditions.

measure	PLC M[SD]	TYR M[SD]	t-value	p-value
HR	-4.54 [7.36]	-4.64 [7.66]	0.08	.936
HRV	20.17 [33.56]	15.34 [33.46]	0.81	.420
PD	0.38 [5.89]	-0.27 [5.68]	0.62	.533
PDV	11.23 [36.36]	11.91 [33.63]	-0.11	.913
SEBR	14.25 [68.83]	6.69 [52.77]	0.69	.494

Note. HR: heart rate, HRV: HR variability, PD: pupil dilation, PDV: variance in PD, SEBR: spontaneous eye blink rate. Columns PLC & TYR depict the mean and standard deviation of the change (in %) from t0 (i.e. baseline) to t1 (i.e. after supplementation) per condition. The last two columns display t-test statistics for comparison between both conditions.
