# Supplementary material for: Catecholamine precursor modulation of human exploration: Evidence from a large gender-balanced sample
Source: PLoS Comput Biol. 2026 Jun 11;22(6):e1014356. doi: 10.1371/journal.pcbi.1014356 (PMC13719043; doi:10.1371/journal.pcbi.1014356)
Supplement: Supplementary file 7 [file pcbi.1014356.s007.pdf]

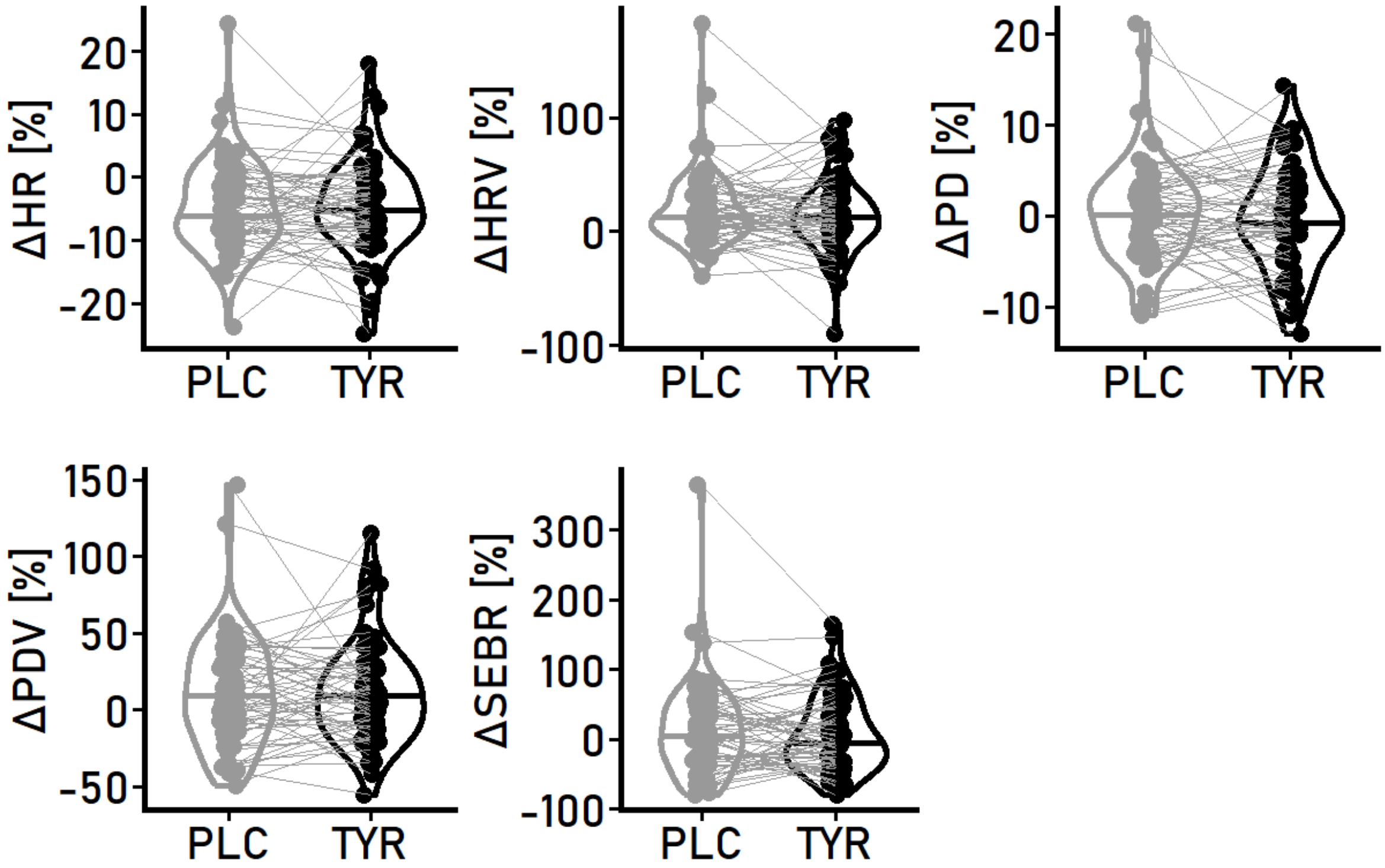

S4 Fig. Changes in Physiological Measures Due to Supplementation.

HR: heart rate, HRV: HR variability, PD: pupil dilation, PDV: variance in PD, SEBR: spontaneous eye blink rate. Y-axes display the change from pre- to post-supplementation (t0 to t1) for both conditions (X-axes).
