# Supplementary material for: Catecholamine precursor modulation of human exploration: Evidence from a large gender-balanced sample
Source: PLoS Comput Biol. 2026 Jun 11;22(6):e1014356. doi: 10.1371/journal.pcbi.1014356 (PMC13719043; doi:10.1371/journal.pcbi.1014356)
Supplement: Supplementary file 8 [file pcbi.1014356.s008.pdf]

**S4 Table. Summary of The Posterior Difference-Distribution**. Parameters denote the group-level means' difference distribution (TYR-PLC) of corresponding SoftMax parameters. β = exploitation (value-based decision-making); Φ = directed exploration (via *bandit heuristic* exploration bonus); ρ = perseveration (stickiness; repetition choice at t-1).

| Parameter | Median | 95% HDI |
|---|---|---|
| $\beta_{diff}$ | 0.01 | [-0.01; 0.019] |
| $\Phi_{diff}$ | -0.13 | [-0.69; 0.451] |
| $\rho_{diff}$ | -0.22 | [-1.97; 1.472] |
