# Supplementary material for: Catecholamine precursor modulation of human exploration: Evidence from a large gender-balanced sample
Source: PLoS Comput Biol. 2026 Jun 11;22(6):e1014356. doi: 10.1371/journal.pcbi.1014356 (PMC13719043; doi:10.1371/journal.pcbi.1014356)
Supplement: Supplementary file 9 [file pcbi.1014356.s009.pdf]

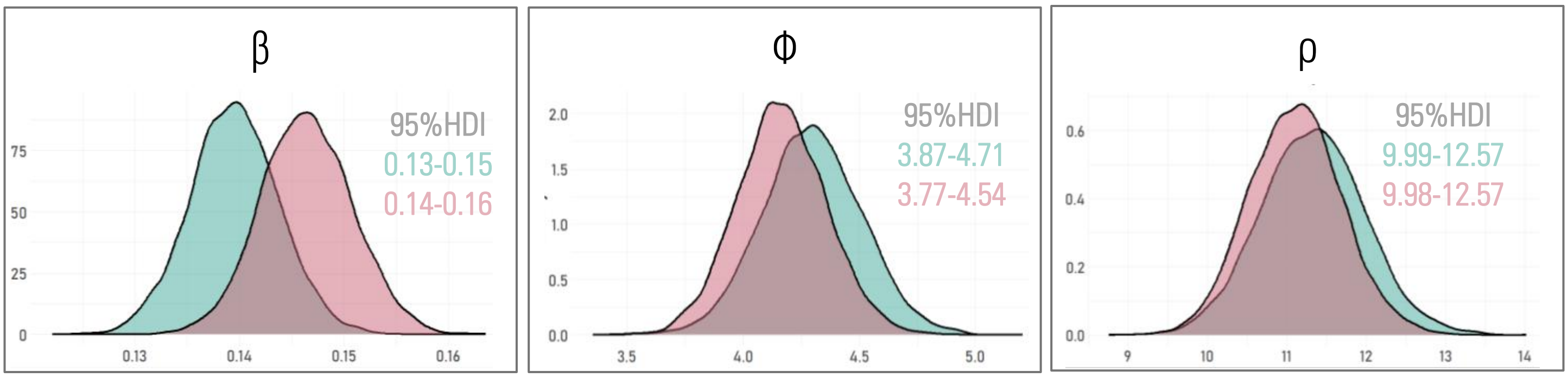

S5 Fig. Posterior Distributions of Group-Level Mean Parameters Derived From the Winning Model (BL + Bandit).
Turquoise = PLC; Magenta = TYR; β = exploitation (value-based decision-making); Φ = directed exploration (via *bandit heuristic* exploration bonus); ρ = perseveration (stickiness; repetition choice at t-1). Numerical ranges in the plots show the 95% HDI for both conditions in their respective colors.
