# Supplementary material for: Catecholamine precursor modulation of human exploration: Evidence from a large gender-balanced sample
Source: PLoS Comput Biol. 2026 Jun 11;22(6):e1014356. doi: 10.1371/journal.pcbi.1014356 (PMC13719043; doi:10.1371/journal.pcbi.1014356)
Supplement: Supplementary file 10 [file pcbi.1014356.s010.pdf]

S5 Table. Correlation of True vs. Recovered Parameter Estimates from Model BL + Bandit.

Simulation	Parameter	Correlation true~fitted
#01		.88
#02		.90
#03	β	.90
#04		.91
#05		.91
#01		.71
#02		.83
#03	ϕ	.79
#04		.74
#05		.77
#01		.89
#02		.87
#03	ρ	.89
#04		.90
#05		.85

Note: All correlation coefficients shown were highly significant ($p<.001$).
