# Supplementary material for: Catecholamine precursor modulation of human exploration: Evidence from a large gender-balanced sample
Source: PLoS Comput Biol. 2026 Jun 11;22(6):e1014356. doi: 10.1371/journal.pcbi.1014356 (PMC13719043; doi:10.1371/journal.pcbi.1014356)
Supplement: Supplementary file 12 [file pcbi.1014356.s012.pdf]

S6 Table. Correlation of True vs. Recovered Parameter Estimates from Model BL+ Bandit+ Habit.

Simulation	Parameter	Correlation true~fitted
#01		.88
#02		.88
#03	β	.89
#04		.84
#05		.85
#01		.43
#02		.75
#03	ϕ	.63
#04		.69
#05		.79
#01		.66
#02		.78
#03	ρ	.75
#04		.55
#05		.81
#01		.77
#02		.64
#03	α_{HOP}	.81
#04		.77
#05		.81

Note: All correlation coefficients shown were highly significant ($p<.001$).
