# Supplementary material for: Catecholamine precursor modulation of human exploration: Evidence from a large gender-balanced sample
Source: PLoS Comput Biol. 2026 Jun 11;22(6):e1014356. doi: 10.1371/journal.pcbi.1014356 (PMC13719043; doi:10.1371/journal.pcbi.1014356)
Supplement: Supplementary file 13 [file pcbi.1014356.s013.pdf]

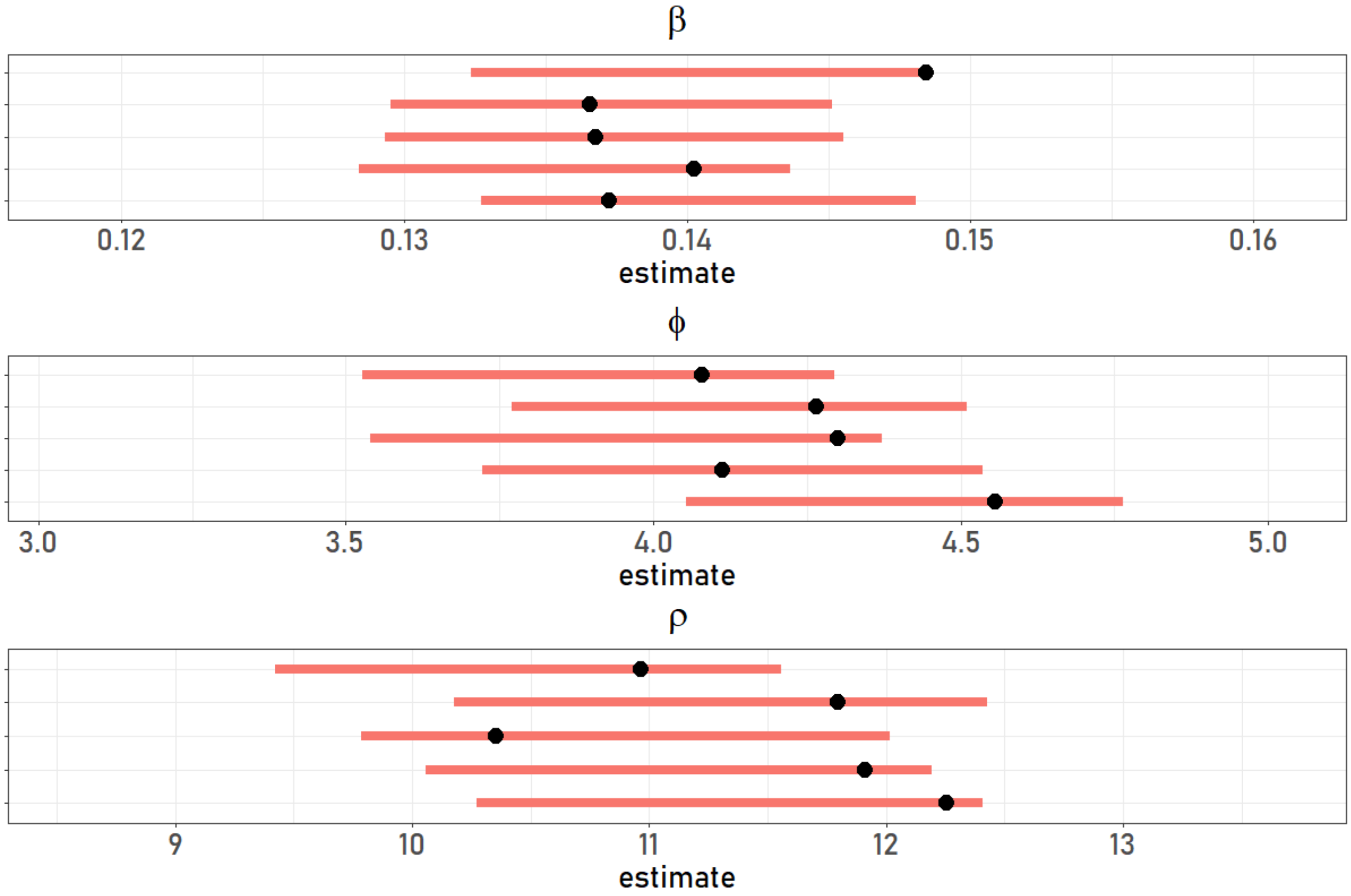

S7 Fig. True and Recovered Group-Level Parameter Estimates Based on Model BL+Bandit. Lines display the 95% HDI from the posterior distribution of group-level parameters representing exploitation (ß), directed-exploration (phi), and FOP (rho) based on the five simulated data sets. Black dots show the true, data generating samples drawn from the original single condition fit (PLC).
