# Supplementary material for: Catecholamine precursor modulation of human exploration: Evidence from a large gender-balanced sample
Source: PLoS Comput Biol. 2026 Jun 11;22(6):e1014356. doi: 10.1371/journal.pcbi.1014356 (PMC13719043; doi:10.1371/journal.pcbi.1014356)
Supplement: Supplementary file 14 [file pcbi.1014356.s014.pdf]

S7 Table. Model Comparison for Model Recovery.

Generating model	Simulation	$elpd_{diff}$ (se_{elpd})	
		KF+EXP+FOP	KF+EXP+HOP
KF + EXP bandit + FOP	#01	-0.6 (3.1)	0.0 (0.0)
	#02	-2.0 (2.8)	0.0 (0.0)
	#03	-6.8 (3.5)	0.0 (0.0)
	#04	-0.9 (3.4)	0.0 (0.0)
	#05	-0.3 (3.4)	0.0 (0.0)
KF + EXP bandit + HOP	#01	**-149.1 (26.6)**	**0.0 (0.0)**
	#02	**-104.4 (20.5)**	**0.0 (0.0)**
	#03	**-194.6 (29.0)**	**0.0 (0.0)**
	#04	**-174.6 (25.4)**	**0.0 (0.0)**
	#05	**-155.7 (27.5)**	**0.0 (0.0)**

Note: generating model: model variant used to simulate choice data (5 data sets each, c.f. Simulations). $elpd_{diff}$ (se_{elpd}): difference in expected log point density and standard error of the difference; Bold cells indicate meaningful differences in model fit, with 0.0 values showing the winning model (i.e. the difference exceeds the SE*2).
