# Supplementary material for: Catecholamine precursor modulation of human exploration: Evidence from a large gender-balanced sample
Source: PLoS Comput Biol. 2026 Jun 11;22(6):e1014356. doi: 10.1371/journal.pcbi.1014356 (PMC13719043; doi:10.1371/journal.pcbi.1014356)
Supplement: Supplementary file 15 [file pcbi.1014356.s015.pdf]

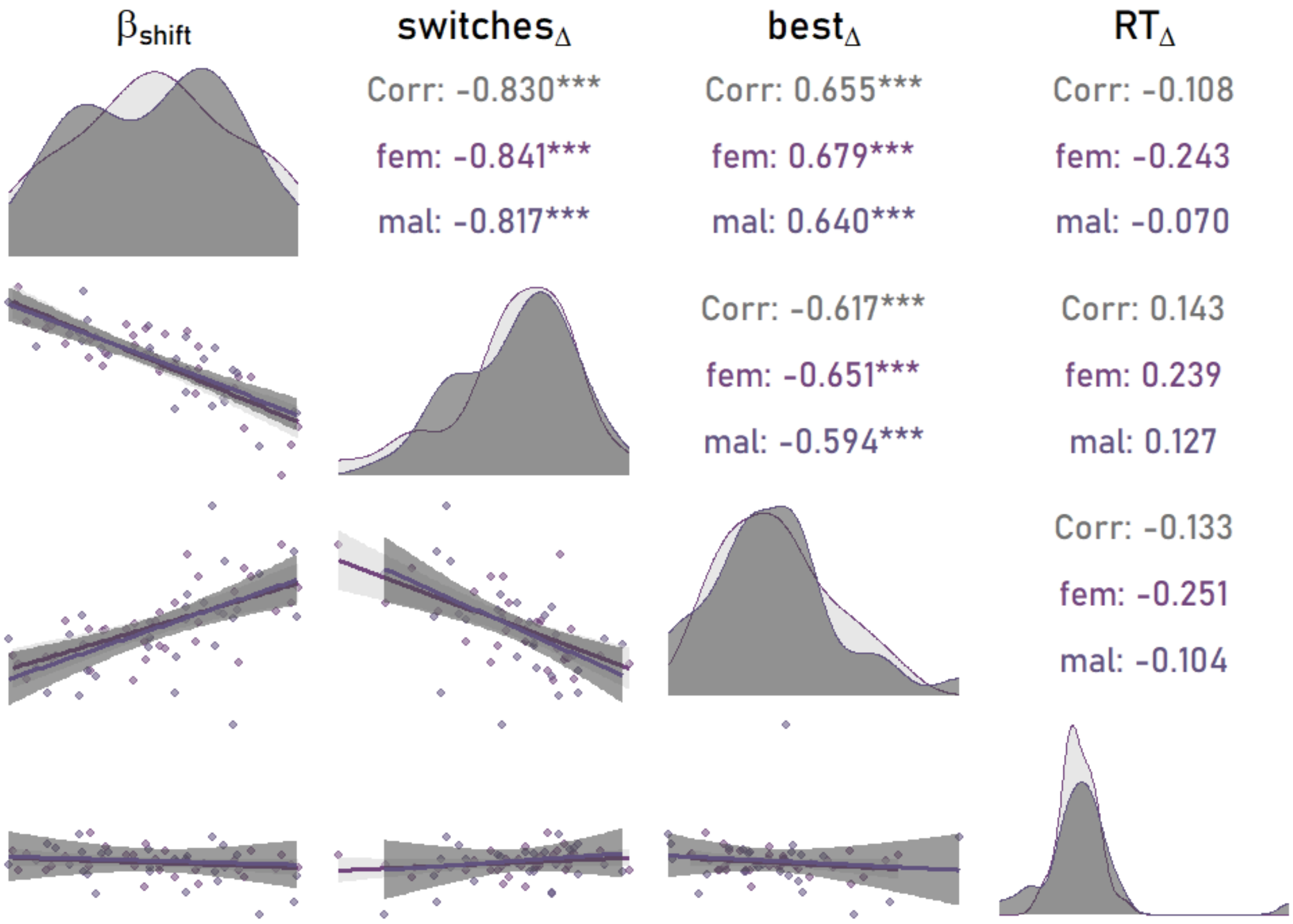

S8 Fig. Association of Task Performance Measures with Supplementation-Effects on Value-Based Decision-Making. $ß_{shift}$: parameter depicting change in exploitation from PLC to TYR; $switches_{\Delta}$: difference in proportion of choice switches between conditions; $best_{\Delta}$: difference in proportion of highest-value choices; RT_{Δ}: difference in median RTs between conditions. The diagonal shows the distribution of the associated parameter/measure separated by gender group. The lower triangular plots depict regression lines, surrounded by standard error intervals (shaded gray areas).
