# Supplementary material for: Catecholamine precursor modulation of human exploration: Evidence from a large gender-balanced sample
Source: PLoS Comput Biol. 2026 Jun 11;22(6):e1014356. doi: 10.1371/journal.pcbi.1014356 (PMC13719043; doi:10.1371/journal.pcbi.1014356)
Supplement: Supplementary file 16 [file pcbi.1014356.s016.pdf]

S8 Table. Posterior Summary Statistics of Group-Level Parameters from Model BL + Bandit + Habit.

	PLACEBO		TYROSINE	
Parameter	Median	95% HDI	Median	95% HDI
α_{HOP}	0.75	[0.71, 0.80]	0.77	[0.74, 0.81]
β	0.12	[0.12, 0.13]	0.13	[0.12, 0.14]
ϕ	5.68	[5.19, 6.21]	5.27	[4.81, 5.73]
ρ	18.14	[16.15, 20.22]	16.48	[14.80, 18.22]
