# Supplementary material for: Catecholamine precursor modulation of human exploration: Evidence from a large gender-balanced sample
Source: PLoS Comput Biol. 2026 Jun 11;22(6):e1014356. doi: 10.1371/journal.pcbi.1014356 (PMC13719043; doi:10.1371/journal.pcbi.1014356)
Supplement: Supplementary file 17 [file pcbi.1014356.s017.pdf]

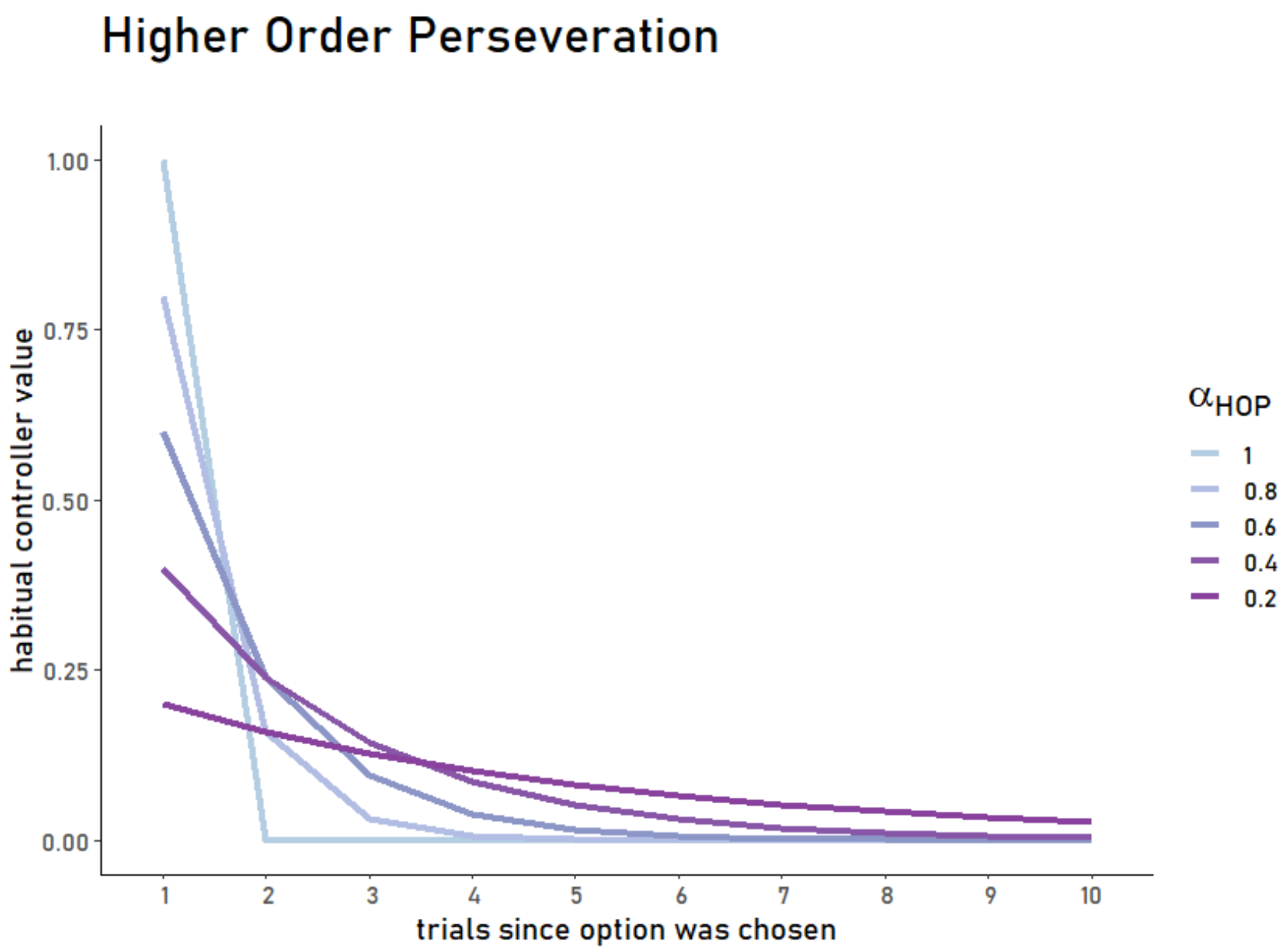

S9 Fig. Exemplary Habitual Controller Values. Shown are habitual controller values for ß=1 and varying decay-rate (α_{HOP}) values.
