# Supplementary material for: Catecholamine precursor modulation of human exploration: Evidence from a large gender-balanced sample
Source: PLoS Comput Biol. 2026 Jun 11;22(6):e1014356. doi: 10.1371/journal.pcbi.1014356 (PMC13719043; doi:10.1371/journal.pcbi.1014356)
Supplement: Supplementary file 18 [file pcbi.1014356.s018.pdf]

S9 Table. Posterior Summary Statistics of TYR-Effect Parameters Based on Model BL + Bandit + Habit Separated by Gender Group.

Group	Parameter	Median	95% HDI	dBF>0 [dBF<0]
FEM	β_{shift}	0.13	[-19.56, 19.54]	1.02 [0.98]
	Φ_{shift}	-1.46	[-19.14, 19.24]	0.80 [1.24]
	ρ_{shift}	-0.97	[-18.75, 20.63]	1.17 [0.85]
	α_{shift}	0.07	[-0.79, 0.29]	1.05 [0.95]
MAL	β_{shift}	0.13	[-19.57, 19.21]	1.01 [0.98]
	Φ_{shift}	-0.85	[-19.80, 19.95]	0.86 [1.15]
	ρ_{shift}	0.15	[-18.22, 19.18]	1.00 [0.99]
	α_{shift}	-0.21	[-0.76, 0.31]	0.91 [1.10]
