# Supplementary material for: Catecholamine precursor modulation of human exploration: Evidence from a large gender-balanced sample
Source: PLoS Comput Biol. 2026 Jun 11;22(6):e1014356. doi: 10.1371/journal.pcbi.1014356 (PMC13719043; doi:10.1371/journal.pcbi.1014356)
Supplement: Supplementary file 19 [file pcbi.1014356.s019.pdf]

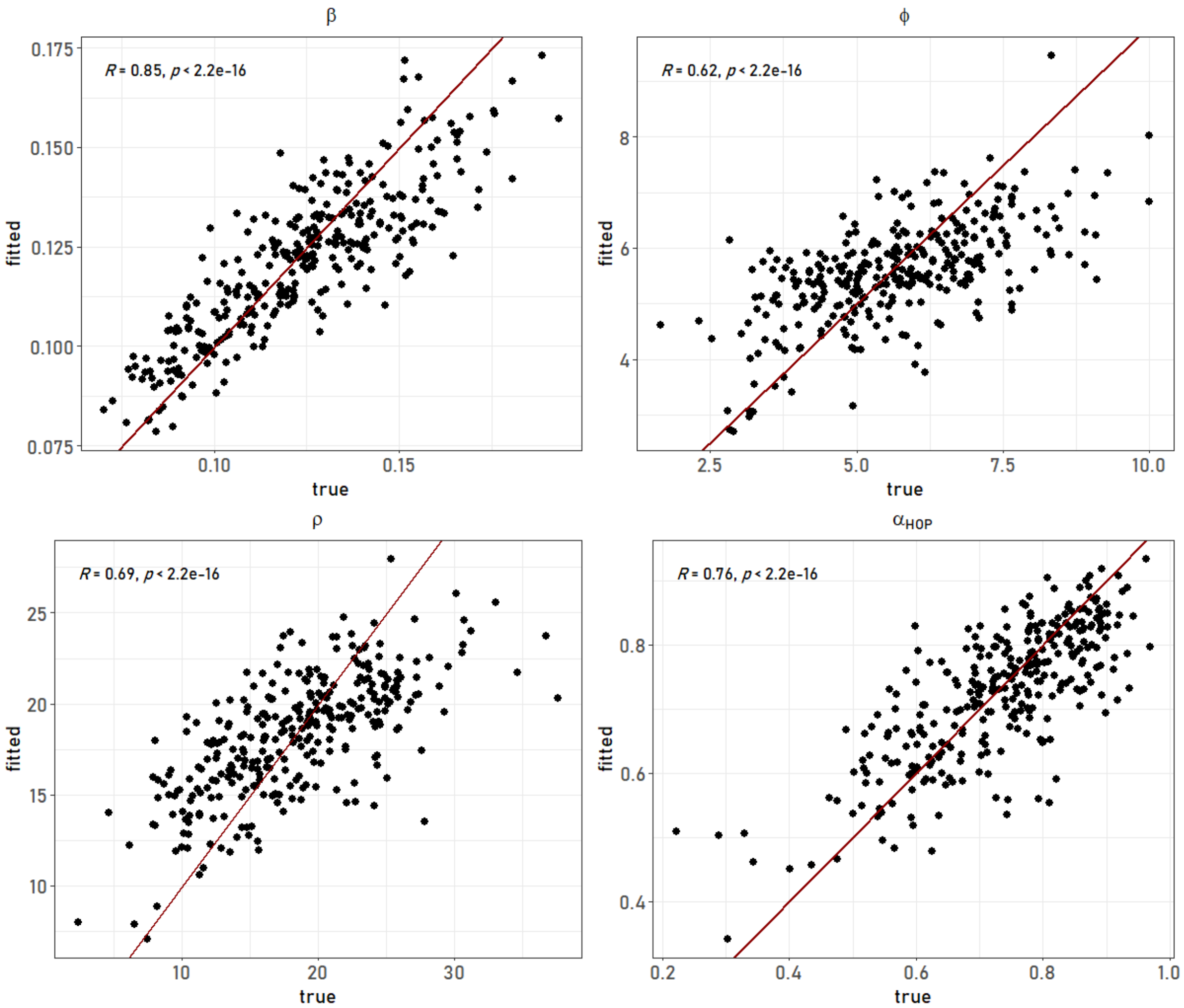

S10 Fig. Parameter Recovery Results from Model BL+ Bandit+ Habit. X-axis: true underlying parameter values used to simulate data for the recovery analysis. Y-Axis: corresponding fitted (i.e. recovered) parameter values. R: correlation coefficient between true and recovered parameters.
