# Supplementary material for: Catecholamine precursor modulation of human exploration: Evidence from a large gender-balanced sample
Source: PLoS Comput Biol. 2026 Jun 11;22(6):e1014356. doi: 10.1371/journal.pcbi.1014356 (PMC13719043; doi:10.1371/journal.pcbi.1014356)
Supplement: Supplementary file 20 [file pcbi.1014356.s020.pdf]

S10 Table. Single Condition Posterior Summary Statistics of DDM Fits.

	PLACEBO		TYROSINE		DIFFERENCE	
Parameter	Median	95% HDI	Median	95% HDI	Median	95% HDI
α	0.82	[0.79, 0.86]	0.83	[0.80, 0.87]	0.010	[-0.04, 0.06]
z	0.56	[0.55, 0.57]	0.57	[0.56, 0.58]	0.008	[-0.01, 0.02]
τ	0.18	[0.17, 0.19]	0.18	[0.17, 0.19]	0.002	[-0.01, 0.01]
δ	-1.48	[-1.69, -1.26]	-1.68	[-1.88, -1.47]	-0.190	[-0.47, 0.12]
