# Supplementary material for: Catecholamine precursor modulation of human exploration: Evidence from a large gender-balanced sample
Source: PLoS Comput Biol. 2026 Jun 11;22(6):e1014356. doi: 10.1371/journal.pcbi.1014356 (PMC13719043; doi:10.1371/journal.pcbi.1014356)
Supplement: Supplementary file 22 [file pcbi.1014356.s022.pdf]

S11 Table. Correlations Between TYR-Effects on BL+ Bandit+Habit Parameters.

Parameter combination	r	95% CI	p-Value
$\alpha_{HOP\text{-}shift} \sim \beta_{shift}$	0.19	[-0.06, 0.42]	.384
$\alpha_{HOP\text{-}shift} \sim \phi_{shift}$	-0.08	[-0.32, 0.17]	.549
$\beta_{shift} \sim \phi_{shift}$	-0.19	[-0.42, 0.06]	.384

Note. $\alpha_{HOP\text{-}shift}$ /β_{shift} /ϕ_{shift}: TYR-effects on parameters depicting HOP step-size, exploitation, and directed exploration. R: Correlation coefficient. 95%CI: 95% confidence interval.
