# Supplementary material for: Catecholamine precursor modulation of human exploration: Evidence from a large gender-balanced sample
Source: PLoS Comput Biol. 2026 Jun 11;22(6):e1014356. doi: 10.1371/journal.pcbi.1014356 (PMC13719043; doi:10.1371/journal.pcbi.1014356)
Supplement: Supplementary file 23 [file pcbi.1014356.s023.pdf]

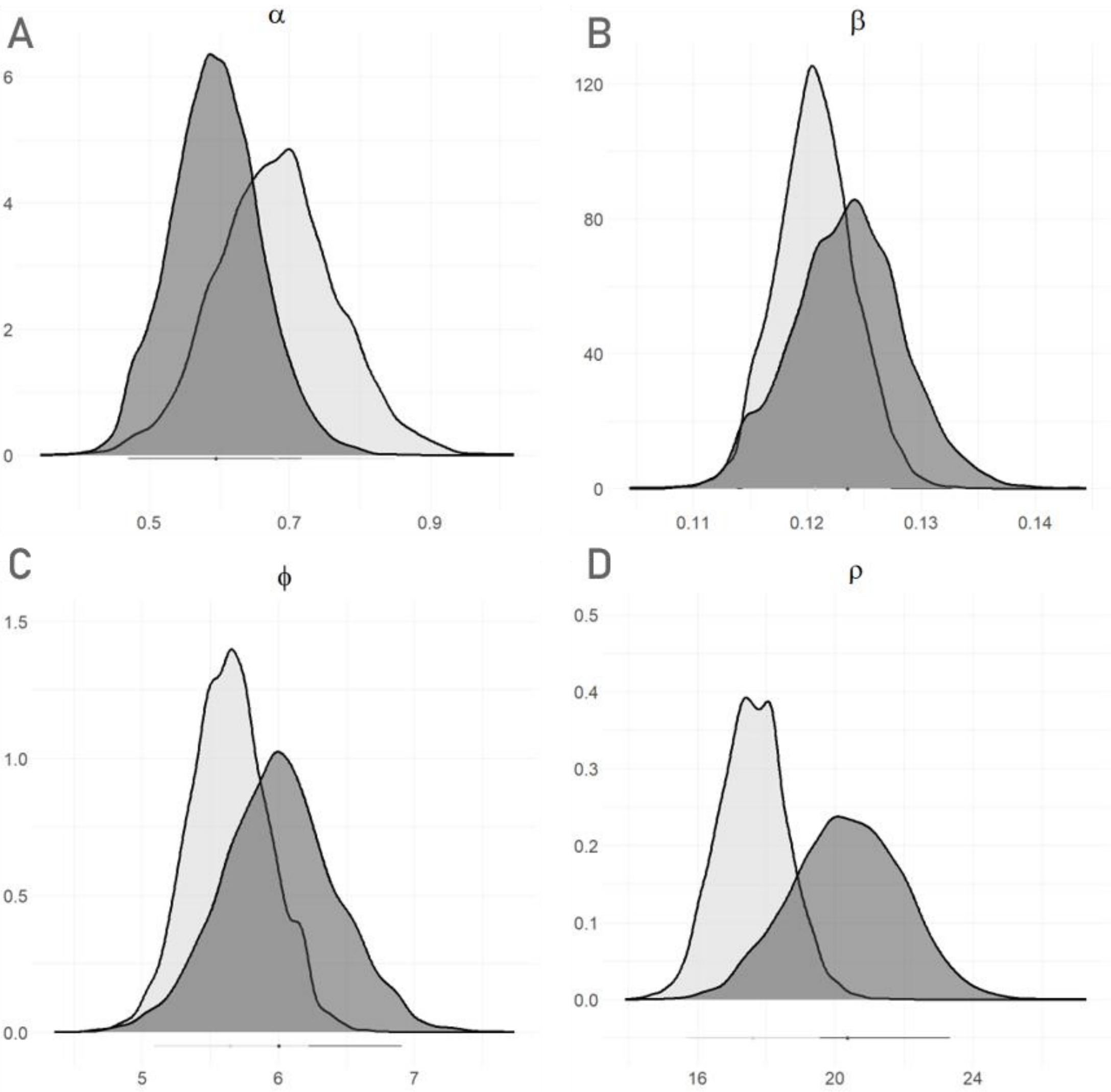

S12 Fig. Posterior Distributions from the Exploratory Model Extension Separated by Gender Group. A) α: decay-rate parameter scaling the influence of past choice history; B) β: SoftMax parameter depicting exploitation; C) Φ: SoftMax parameter depicting directed exploration (via bandit-heuristic); D) ρ: SoftMax parameter depicting perseveration. Parameters were derived from the exploratory model extension of the winner model (BL + Bandit) by accounting for habitual perseveration (*higher order perseveration*). Light gray: female; Dark gray: male. Lines on the x-aches indicate the 95% HDI for each gender group in their respective colors.
