# Supplementary material for: Catecholamine precursor modulation of human exploration: Evidence from a large gender-balanced sample
Source: PLoS Comput Biol. 2026 Jun 11;22(6):e1014356. doi: 10.1371/journal.pcbi.1014356 (PMC13719043; doi:10.1371/journal.pcbi.1014356)
Supplement: Supplementary file 24 [file pcbi.1014356.s024.pdf]

S12 Table. Difference in Physiological Changes Between Conditions for Male Subsample.

measure	PLC<br>M[SD]	TYR<br>M[SD]	t-value	p-value
HR	-5.94 [6.45]	-3.63 [7.34]	-1.46	.155
HRV	20.89 [30.73]	14.00 [35.96]	0.92	.362
PD	-0.06 [6.22]	-0.50 [5.75]	0.35	.728
PDV	12.47 [33.67]	5.24 [31.85]	1.51	.142
SEBR	19.03 [85.61]	8.31 [54.81]	0.82	.418

Note. HR: heart rate, HRV: HR variability, PD: pupil dilation, PDV: variance in PD, SEBR: spontaneous eye blink rate. Columns PLC & TYR depict the mean and standard deviation of the change (in %) from t0 (i.e. baseline) to t1 (i.e. after supplementation) per condition. The last two columns display t-test statistics for comparison between both conditions.
