# Supplementary material for: Catecholamine precursor modulation of human exploration: Evidence from a large gender-balanced sample
Source: PLoS Comput Biol. 2026 Jun 11;22(6):e1014356. doi: 10.1371/journal.pcbi.1014356 (PMC13719043; doi:10.1371/journal.pcbi.1014356)
Supplement: Supplementary file 25 [file pcbi.1014356.s025.pdf]

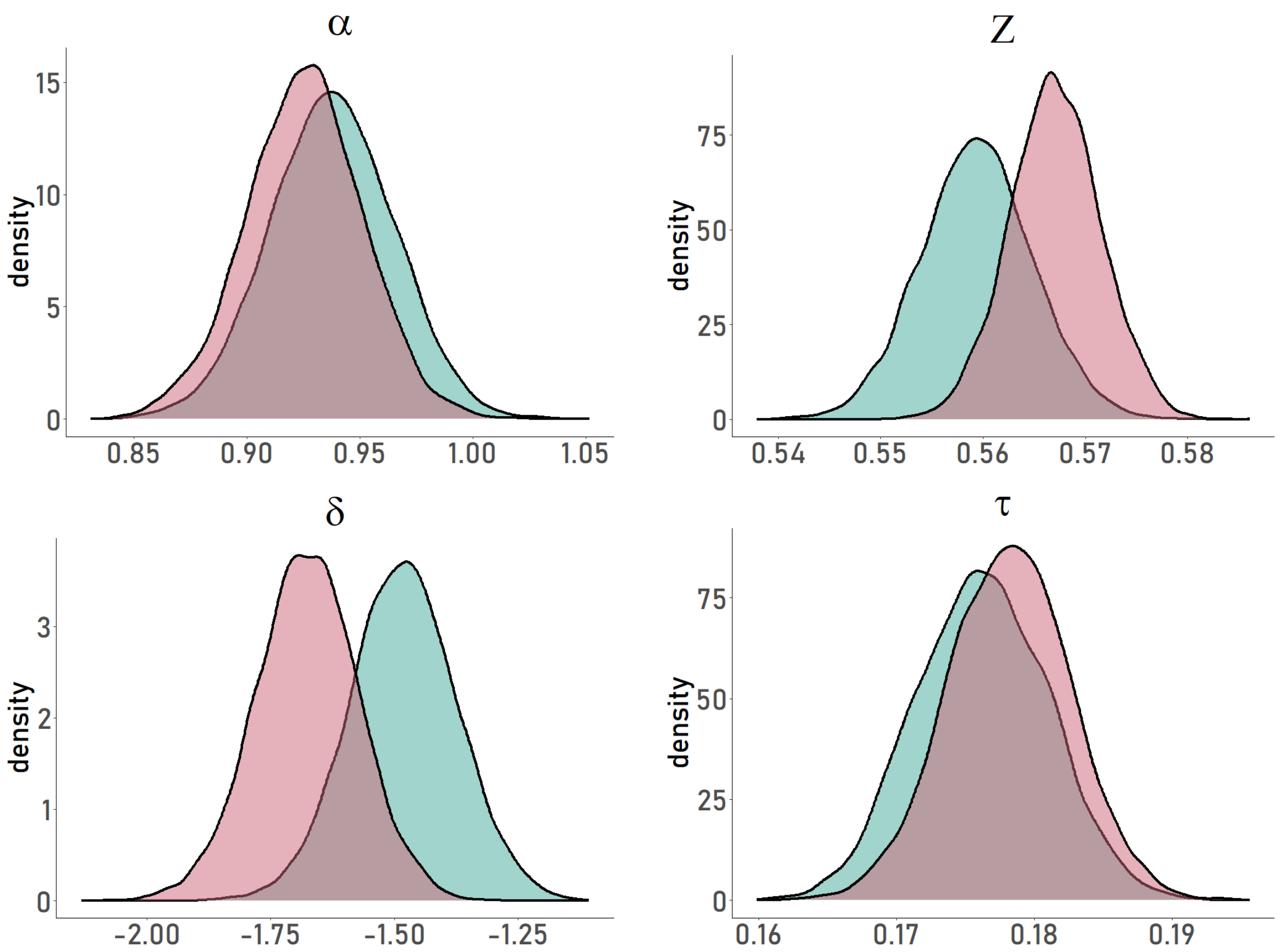

S13 Fig. Posterior Distributions of DDM-Parameters Separated by Condition. Turquoise = PLC; magenta = TYR; α = boundary separation; z = bias; δ = drift rate; τ = non-decision time. Decision Boundaries were defined as 0 = stay (i.e. repetition of preceding choice); 1= switch (choice alteration).
