# Supplementary material for: Catecholamine precursor modulation of human exploration: Evidence from a large gender-balanced sample
Source: PLoS Comput Biol. 2026 Jun 11;22(6):e1014356. doi: 10.1371/journal.pcbi.1014356 (PMC13719043; doi:10.1371/journal.pcbi.1014356)
Supplement: Supplementary file 26 [file pcbi.1014356.s026.pdf]

S13 Table. Control Regression Models Predicting Model-Agnostic Performance Indices While Accounting for Weight and Age.

	%best choice			*RT*			*%switches*		
**Predictors**	Estimate	CI	p	Estimate	CI	p	Estimate	CI	p
**(Intercept)**	6.60×10^{-1} ***	0.65 – 0.67	**<0.001**	3.40×10^{-1} ***	0.32 – 0.36	**<0.001**	3.29×10^{-1} ***	0.30 – 0.36	**<0.001**
**cond [tyr]**	1.63×10^{-2} *	0.00 – 0.03	**0.019**	4.30×10^{-3}	0.00 – 0.01	**0.045**	-2.39×10^{-2}***	-0.04 – -0.01	**<0.001**
**gend [mal]**	2.50×10^{-3}	-0.02 – 0.02	0.814	-2.73×10^{-3}	-0.03 – 0.02	0.838	-4.65×10^{-2} *	-0.09 – -0.00	**0.029**
**cond [tyr] * gend [mal]**	-4.69×10^{-3}	-0.02 – 0.01	0.640	2.34×10^{-3}	-0.00 – 0.01	0.449	4.72×10^{-3}	-0.01 – 0.02	0.623

** p<0.05 ** p<0.01 *** p<0.001 ; cond = condition (plc vs. tyr); gend =self-identified gender (female vs. male)*
