# Supplementary material for: Catecholamine precursor modulation of human exploration: Evidence from a large gender-balanced sample
Source: PLoS Comput Biol. 2026 Jun 11;22(6):e1014356. doi: 10.1371/journal.pcbi.1014356 (PMC13719043; doi:10.1371/journal.pcbi.1014356)
Supplement: Supplementary file 27 [file pcbi.1014356.s027.pdf]

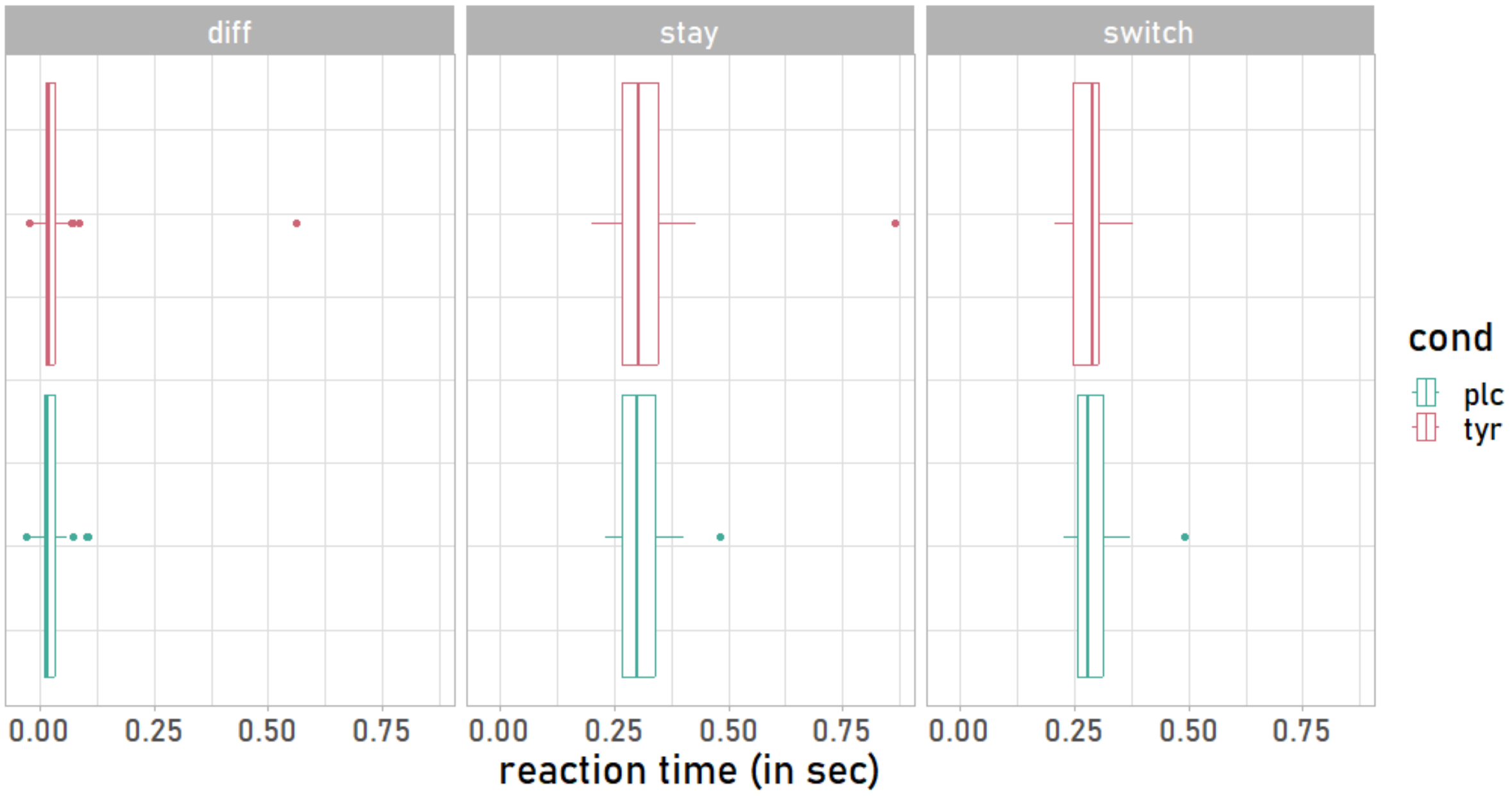

S14 Fig. Mean Reaction Times For Stay vs. Switch Decisions. Panels *stay* and *switch* depict mean RTs for choice repetitions, and alterations, respectively. The first panel shows the difference in RTs between these choice types. Inner lines indicate the median, while outer boxes indicate quartiles. Turquise/magenta signify supplementation conditions placebo (plc) and tyrosine (tyr), respectively.
