# Supplementary material for: Catecholamine precursor modulation of human exploration: Evidence from a large gender-balanced sample
Source: PLoS Comput Biol. 2026 Jun 11;22(6):e1014356. doi: 10.1371/journal.pcbi.1014356 (PMC13719043; doi:10.1371/journal.pcbi.1014356)
Supplement: Supplementary file 28 [file pcbi.1014356.s028.pdf]

S14 Table. Overview of Self-Report Instruments.

Construct	Instrument	Reference
Alcohol Consumption	Alcohol Use Disorder Identification Test (AUDIT)	Saunders et al. (1993)
Impulsivity	Barratt Impulsivity Scale (BIS15)	Patton et al., (1995)
Depression	Beck's Depression Inventory (BDI)	Beck et al. (1996)
Reward-Related Behaviour	Behavioural Inhibition Scale & Behavioural Activation Scale (BIS/BAS)	Carver & White (1994)
Time Perception	Zimbardo Time Perspective Inventory (ZTPI)	Zimbardo & Boyd (1996)
