# Supplementary material for: Catecholamine precursor modulation of human exploration: Evidence from a large gender-balanced sample
Source: PLoS Comput Biol. 2026 Jun 11;22(6):e1014356. doi: 10.1371/journal.pcbi.1014356 (PMC13719043; doi:10.1371/journal.pcbi.1014356)
Supplement: Supplementary file 29 [file pcbi.1014356.s029.pdf]

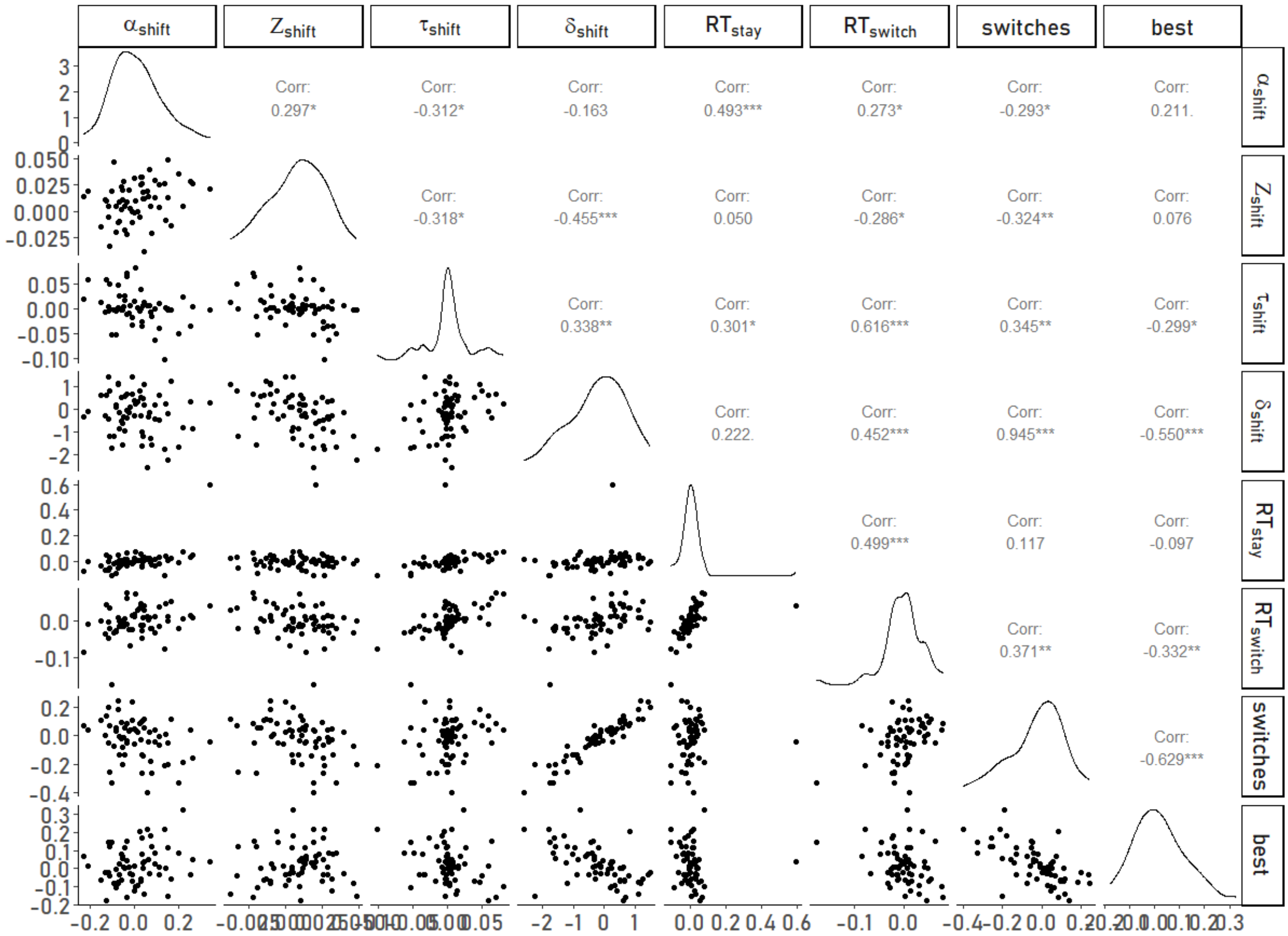

S15 Fig. Correlations Between DDM-Parameters and Model-Agnostic Measures. "shift" parameters represent the TYR-effect on DDM-parameters. RT switch/stay denote median RTs for switch/stay choices. Switches and Best refer to the model agnostic measures proportion of switch trials and proportion of optimal (max. reward) choices, respectively.
