# Supplementary material for: Catecholamine precursor modulation of human exploration: Evidence from a large gender-balanced sample
Source: PLoS Comput Biol. 2026 Jun 11;22(6):e1014356. doi: 10.1371/journal.pcbi.1014356 (PMC13719043; doi:10.1371/journal.pcbi.1014356)
Supplement: Supplementary file 30 [file pcbi.1014356.s030.pdf]

S15 Table. Priors for DDM Hyper-Parameters. For each subject-level parameter (first coloumn), M & SD denote priors for corresponding group-level parameters. Note that the bias parameter z was transformed to standard normal space within the model, so that priors listed below correspond to the untransformed parameter estimates.

Parameter	M	SD
α	uniform (0.001, 5)	uniform (0.001, 10)
z	normal (0, 10)	uniform (0, 10)
τ	uniform (0.001, 2)	uniform (0.001, 10)
δ	uniform (-5, 5)	uniform (0.001, 10)
